# The Pu.1 target gene Zbtb11 regulates neutrophil development through its integrase-like HHCC zinc finger

**DOI:** 10.1038/ncomms14911

**Published:** 2017-04-06

**Authors:** Maria-Cristina Keightley, Duncan P. Carradice, Judith E. Layton, Luke Pase, Julien Y. Bertrand, Johannes G. Wittig, Aleksandar Dakic, Andrew P. Badrock, Nicholas J. Cole, David Traver, Stephen L. Nutt, Julia McCoey, Ashley M. Buckle, Joan K. Heath, Graham J. Lieschke

**Affiliations:** 1 Australian Regenerative Medicine Institute, Monash University, Clayton, Victoria 3800, Australia; 2 The Walter and Eliza Hall Institute of Medical Research, 1G Royal Parade, Parkville, Victoria 3050, Australia; 3 Department of Medical Biology, University of Melbourne, Parkville, Victoria 3010, Australia; 4 Ludwig Institute for Cancer Research, Melbourne-Parkville Branch, The Royal Melbourne Hospital, Parkville, Victoria 3050, Australia; 5 Department of Pathology and Immunology, University of Geneva—CMU, 1211 Geneva 4, Switzerland; 6 Faculty of Life Sciences, The University of Manchester, Manchester M13 9PL, UK; 7 Motor Neuron Disease Research Group, Faculty of Medicine and Health Sciences, Macquarie University, Sydney, New South Wales 2109, Australia; 8 Department of Cellular and Molecular Medicine, University of California at San Diego, La Jolla, California 92093, USA; 9 Department of Biochemistry and Molecular Biology, Monash University, Clayton, Victoria 3800, Australia

## Abstract

In response to infection and injury, the neutrophil population rapidly expands and then quickly re-establishes the basal state when inflammation resolves. The exact pathways governing neutrophil/macrophage lineage outputs from a common granulocyte-macrophage progenitor are still not completely understood. From a forward genetic screen in zebrafish, we identify the transcriptional repressor, ZBTB11, as critical for basal and emergency granulopoiesis. ZBTB11 sits in a pathway directly downstream of master myeloid regulators including PU.1, and *TP53* is one direct ZBTB11 transcriptional target. *TP53* repression is dependent on ZBTB11 cys116, which is a functionally critical, metal ion-coordinating residue within a novel viral integrase-like zinc finger domain. To our knowledge, this is the first description of a function for this domain in a cellular protein. We demonstrate that the PU.1–ZBTB11–TP53 pathway is conserved from fish to mammals. Finally, Zbtb11 mutant rescue experiments point to a ZBTB11-regulated TP53 requirement in development of other organs.

## Abstract (web summary)

Neutrophils are increased in response to injury and infection but how they form from a common granulocyte-macrophage progenitor is unclear. Here, the authors identify a role for the transcriptional repressor ZBTB11 in zebrafish, which is regulated by master myeloid regulators and represses *TP53*.

Trillions of neutrophils are required every day for host defence. In response to threats like infection and injury, the neutrophil population must be rapidly expanded^1^. Maintaining steady-state production of short-lived, terminally differentiated neutrophils and rapidly increasing their production on demand requires tightly coordinated but flexible regulation^2^,^3^. Neutrophil expansion relies on haemopoietic stem cell (HSC)-derived common myeloid progenitors that can give rise to macrophages as well as neutrophils^4^. Lineage choice is determined by complex interplay of transcription factor regulatory networks^5^,^6^. It is thought that low levels of Pu.1 contribute to development along the neutrophil lineage, with contributions by C/ebpα and Gfi1, whereas high levels of Pu.1 together with Irf8 direct development along the macrophage lineage. This occurs within the context of integral signalling pathways, cytokines and epigenetic modifiers that act in concert with transcription factors to regulate haemopoietic output. Despite intensive study in this area, the precise mechanisms governing neutrophil specification are still not known.

TP53 has long been synonymous with its central role as a tumour suppressor and protector of genome integrity. Its functional sphere of influence extends beyond this role, however, and TP53 has a key role in HSC development. High levels of TP53 are associated with HSC quiescence and expression of TP53 must be downregulated in order for HSCs to exit quiescence and enter the cell cycle^7^,^8^. A hypomorphic Mdm2 allele, which results in high levels of Tp53, causes profound lymphopenia and a decrease in granulocytes to 60% of wild-type (WT) value^9^, suggesting a differential developmental requirement for tightly regulated Tp53 across haemopoietic lineages.

Zbtb11 (Zinc finger and BTB (broad-complex, tramtrack, bric-à-brac) domain containing 11) is an enigmatic member of the ZBTB (BTB-ZF or POK—Pox virus and Krüppel-like zinc fingers) superfamily of ∼49 proteins characterized by the family defining protein–protein interacting BTB domain and varying numbers of zinc fingers^10^,^11^,^12^. The BTB domain in these proteins can homodimerize or heterodimerize with cell-specific protein partners including corepressors such as histone deacetylases (HDACs) and a variable number of zinc fingers can mediate DNA binding. Most ZBTB proteins are transcriptional repressors, components of large multi-protein corepressor complexes that bind target promoters and repress transcription. A minority of ZBTB proteins can act as transcriptional activators, highlighting their cell context-dependent potential for specificity^13^,^14^. Several ZBTB proteins have important roles in haemopoiesis^11^,^12^ and oncogenic roles in promyelocytic leukaemia^15^,^16^. Previous observations have correlated high ZBTB11 expression with human myeloid lineage cells and several subtypes of acute myeloid leukaemia^17^,^18^. Originally identified as a regulator of metallothionein 2A 18 years ago^19^, little else is known about Zbtb11 and its biological function.

Herein we define a biological function for Zbtb11 and provide the first functional evidence for its role in myeloid lineage development. We describe a new evolutionarily conserved genetic and biochemical pathway connecting the master myeloid regulator PU.1 and ZBTB11 in basal neutrophil development and emergency granulopoiesis, and show that early in haemopoiesis Zbtb11 is required for neutrophil but not macrophage development. We identify ZBTB11 as a new transcriptional repressor of *TP53*, pin-pointing key residues that are required for ZBTB11 function as determinants of a novel N-terminal HHCC (His, His, Cys, Cys) zinc finger and establish a Zbtb11–Tp53-dependent pathway that regulates neuronal cell death.

## Results

### *Marsanne* is a *Zbtb11* allele with a deficit in neutrophils

**Figure 1:**
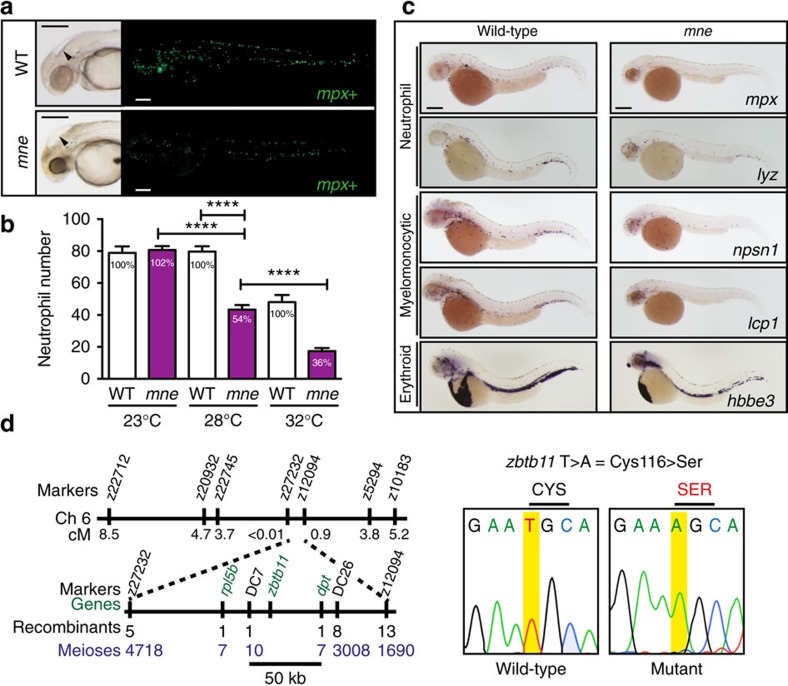
*mne* presents a myeloid phenotype at 48 h.p.f. and maps to *zbtb11.* (**a**) Brightfield: enlarged fourth ventricle (arrow-head), small dark eye and opacity due to CNS degeneration. Fluorescence: fewer *mpx:EGFP*-positive cells in *mne* compared to WT (enumerated in **b**). (**b**) Neutrophil deficiency, reflected by the abundance of *mpx:EGFP*-positive cells, is exacerbated by increasing temperature in the temperature-sensitive mutant *mne*. Percentage of neutrophils compared to WT is stated in the columns (data from one experiment (from left to right: *n*=11, 12, 19, 12, 10, 11) representative of four biologically independent replicates, two-tailed *t*-test; ****P≤0.0001). (**c**) Decreased expression of multiple myeloid genes in *mne*. Whole-mount *in situ* hybridization of *mne* and WT siblings with neutrophil (*mpx*, *lyz*), myelomonocytic (*lcp1*, *npsn1*), and erythroid (*hbbe3*) markers at 48 h.p.f. (**d**) Summary of *marsanne* genome scan data defining a region narrowed by positional cloning to a 50 kb critical interval, which contained a single gene, *zbtb11.* Sequencing of *mne zbtb11* identifies a single T>A transversion in exon 2 resulting in a Cys>Ser substitution at amino acid 116 (C116S); scale bars, 200 μm (**a**,**c**).

To reveal new regulators required for myelopoiesis, we undertook a forward genetic screen in ethylnitrosourea-mutagenized zebrafish, and identified *marsanne* (*mne*) as a temperature-sensitive mutant with defective myeloid development evidenced by a deficit of cells expressing the neutrophil markers *myeloperoxidase* (*mpx*) and *lysozyme* (*lyz*), and myelomonocytic markers *nephrosin* (*nspn1*) and *l-plastin* (*lcp1*) (Fig. 1a–c; Supplementary Fig. 1). A similar decrease in cell number across multiple myeloid lineage genes pointed to a defect in the number of neutrophils and not just aberrant expression of *mpx* (Fig. 1c). Erythromyeloid progenitors are specified normally in *mne* and expression of erythroid genes (for example, embryonic haemoglobin (*hbbe3* and *O*-dianisidine staining)) is preserved for the life of the embryo (5 days post fertilization (d.p.f.)), localizing the haemopoietic defect to the myeloid compartment (Fig. 1c; Supplementary Fig. 2).

The *mne* mutation was positionally cloned and found to be a T→A transversion resulting in a Cys to Ser change at amino acid 116 in the N-terminal domain of Zbtb11 (Fig. 1d). The temperature sensitivity of the mutation indicated that the allele was hypomorphic and the deficit in neutrophils could either be augmented (33 °C) or ameliorated (21 °C) depending on the temperature at which *mne* mutants were raised (Fig. 1b; Supplementary Fig. 1). Standard genetic proofs including morpholino-mediated phenocopy, *mne* rescue by overexpression of WT but not mutant (C116S) Zbtb11 and independent non-complementing CRISPR/Cas9-generated indel *zbtb11* alleles validated the positional cloning (Supplementary Fig. 3). Since a biological function had not previously been ascribed to Zbtb11 we set out to characterize its expression and function using genetic mutants and biochemistry.

### *mne* has impaired neutrophil development and differentiation

**Figure 2:**
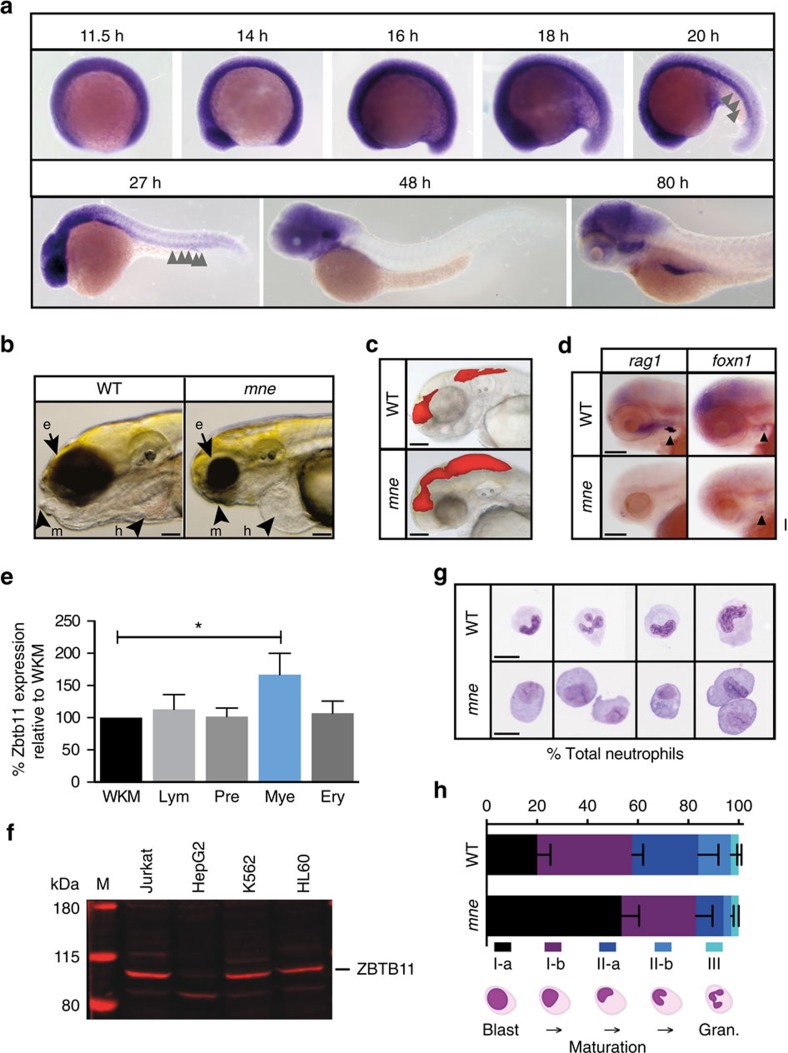
Zbtb11 expression and *mne* phenotype including delayed neutrophil maturation. (**a**) Whole-mount *in situ* hybridization (WISH) showing widespread expression of Zbtb11 in the developing embryo up until 19 h.p.f., which becomes progressively restricted up until 80 h.p.f. Arrows indicate Zbtb11 expression in the intermediate cell mass (ICM). (**b**) At 96 h.p.f., *mne* exhibits ocular, craniofacial and cardiovascular defects. e, eye; h, heart; m, mandibular cartilage. (**c**) Injection of rhodamine at 48 h.p.f. shows enlarged dye volume in fourth ventricle in *mne* compared to WT. (**d**) Loss of *rag1* expression in *mne* at 82 h.p.f. compared to WT. *Foxn1* marking the thymic primordium is expressed in *mne* and WT. (**e**) RT–qPCR of Zbtb11 expression in FACS sorted adult zebrafish blood cell populations (mean±s.d.; *P≤0.05; *n*=1 experiment; triplicate replicates on cDNA isolated from purified haemopoietic populations derived from pooled kidney marrows). Ery, erythroid; Lym, lymphoid; Mye, myeloid; Pre, precursors; WKM, whole kidney marrow; Mann–Whitney test. (**f**) Immunoblot showing ZBTB11 is expressed in human myeloid and lymphoid cell lines and with lower expression in HepG2 hepatocytes (50 μg protein per lane); M, protein ladder with molecular weight in kDa as indicated. (**g**) Examples of FACS-sorted neutrophils from *mne* and WT following May–Grünwald Giemsa staining. (**h**) Quantification of neutrophil sub-populations in *mne* and WT according to maturity shown as percentage of total cells counted. Schema below graph defines how sub-populations were scored. Gran., granulocytes. *n*=3 biologically independent experiments (mean±s.e.m.); (**a**) whole embryo scale^70^; scale bars, 200 μm (**b**–**d**,**g**).

Zbtb11 is maternally deposited and then widely expressed early in development (Fig. 2a). After 24 hours post fertilization (h.p.f.), its expression wanes at many sites but is retained in the nervous system. Consistent with its expression pattern, *mne* also has a multisystem embryonic lethal phenotype including impaired craniofacial development (Fig. 2b) and hydrocephalus (Figs 1a and 2c). Injection of rhodamine dye into the fourth ventricle clearly shows its enlargement in *mne* compared to WT (Fig. 2c). However, early in haemopoietic development when primitive haemopoiesis prevails, *mne* displays a highly specific, lineage-restricted, myeloid phenotype. Consistent specifically with the myeloid-failure phenotype of *mne*, Zbtb11 is expressed in the zebrafish haemopoietic intermediate cell mass (Fig. 2a). Several pointers indicate an ongoing requirement for Zbtb11 in sustaining definitive haemopoiesis. By 5 d.p.f., when there is strong local expression of *rag1*-expressing T-cells in the thymus in the WT, *mne* lacks *rag1* expression in the thymus despite development of the thymic primordia as marked by *foxn1* (Fig. 2d). Thrombocyte numbers are also reduced by 82 h.p.f. (Supplementary Fig. 2e). Despite normal specification of HSCs, as defined by cells expressing *runx1* and *myb* along the ventral wall of the dorsal aorta (Supplementary Fig. 2b), *myb* expression is absent in *mne* caudal haemopoietic tissue at 72 and 96 h.p.f., suggesting that maintenance of the stem cell pool is also disrupted in *mne* (Supplementary Fig. 2c,d). This indicates a broader failure to sustain definitive haemopoiesis later in development and highlights the sensitivity of granulocytes as the first lineage affected in *mne*. PCR with reverse transcription (RT–PCR) of fluorescence-activated cell sorting (FACS)-sorted adult zebrafish kidney marrow confirms expression of Zbtb11 in adult haemopoietic cells, with highest levels in myeloid cells (Fig. 2e). Consistent with public domain RNA expression profiles^17^,^18^, we have confirmed that ZBTB11 protein is highly expressed in human Jurkat (T cells), K562 cells (a BCR-ABL positive blast crisis erythroleukaemia) and HL60 (promyelocytic leukaemia) cells (Fig. 2f). Lower expression in HepG2 liver cancer cells correlates with hepatocellular carcinoma expression profiling showing very low ZBTB11 expression^20^. FACS-sorted embryonic neutrophils from *mne* and WT stained with May-Grünwald Giemsa exhibited an abnormally higher proportion of immature neutrophils in *mne*. Hence, there is both a quantitative and qualitative myeloid development defect as a result of Zbtb11 dysfunction (Fig. 2g,h).

### Zbtb11 is required for basal and emergency granulopoiesis

**Figure 3:**
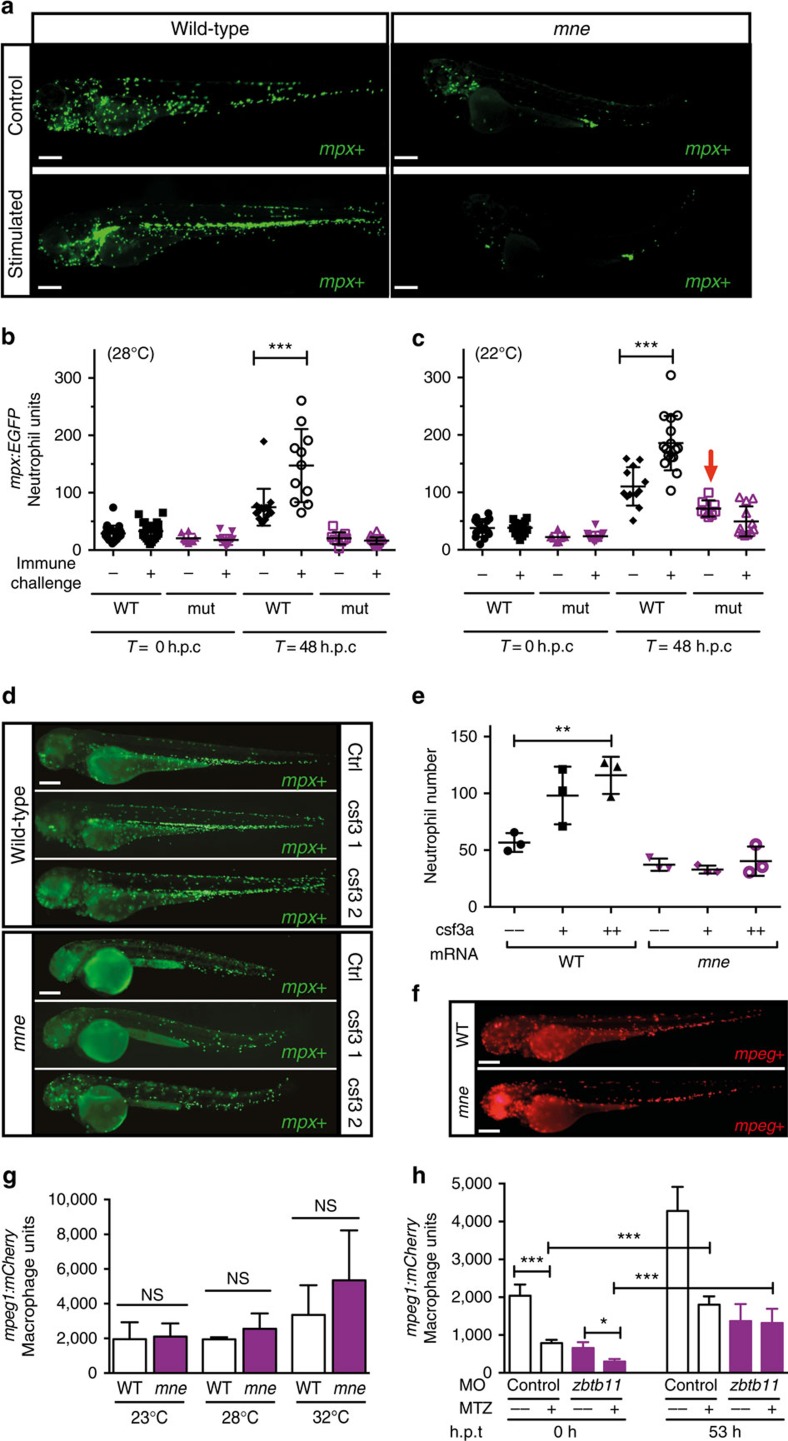
Zbtb11 deficiency results in failure of emergency granulopoiesis. (**a**) Fluorographs of representative embryos either unstimulated (control) or 48 h post challenge with frozen *T. marneffei* spores. (**b**) Graph showing enumeration of neutrophils in embryos raised at 28 °C; data from one representative experiment of three biological replicates; each point represents one embryo (from left to right: *n*=21, 24, 8, 13, 16, 11, 9, 20); mean ±s.d., Mann–Whitney test; ***P≤0.001; h.p.c., hours post challenge with frozen *T. marneffei* spores; (**c**) Enumeration of neutrophils raised at 22 °C (arrow indicates the number of unstimulated neutrophils in *mne* approaches that of WT at 22 °C); details as for (**b**) (from left to right: *n*=15, 16, 9, 13, 12, 16, 8, 12); one-tailed *t*-test; ***P≤0.001. (**d**) Overexpression of csf3a (G-CSF) results in vigorous stimulation of neutrophil expansion in WT but not *mne* embryos shown as *mpx-EGFP^+^* fluorescent neutrophils; ctrl, control; csf3 1 and csf3 2, 0.05 ng and 0.1 ng of csf3a mRNA, respectively. (**e**) Enumeration of neutrophils in **d**. Mean ±s.e.m.; two-tailed *t*-test; *n*=3 biologically independent experiments; **P≤0.01). (**f**) Fluorographs of representative WT Tg(*mpeg1:mCherry*) and *mne;mpeg1:mCherry* embryos showing similar numbers of macrophages. (**g**) Enumeration of macrophages in **f** shows that at 48 h.p.f. macrophage development remains unaffected regardless of severity of *marsanne* phenotype. *n*=2 biologically independent experiments; mean ±s.e.m., two-tailed *t*-test; NS=*P*>0.05. (**h**) Macrophage development is independent of Zbtb11. Repopulation of macrophages following their selective ablation by metronidazole (MTZ) treatment of Tg(*mpeg1:Gal4FF/UAS:nfsb-mCherry/mpx:EGFP*) embryos occurs in both Zbtb11 and control morphants. Details as for **b** (from left to right: *n*=18, 18, 7, 12, 14, 16, 6, 12); *P≤0.05; ***P≤0.001; h.p.t., hours post treatment; scale bars, 200 μm (**a**,**d**,**f**).

The *mne zbtb11* allele is temperature-sensitive, permitting the severity of the phenotype to be altered by temperature shifts, providing a gradient of low to high phenotypic severity with increasing temperature. The neutrophil depletion phenotype correlated strongly with an increasingly overall severe phenotype in *mne* compared to WT (Supplementary Fig. 1a,b). As the neutrophil deficiency in *mne* was not absolute, we examined if stimulation of granulopoiesis could overcome the defect. Freeze-killed *T. marneffei* fungal spores were injected as a global microbial stimulus of granulopoiesis, resulting in strong augmentation of the neutrophil population size in WT embryos, but no rescue of the granulopoietic defect in *mne* (Fig. 3a,b). Even when Zbtb11 function and basal neutrophil numbers were partially restored by exploiting the temperature-sensitive *mne* allele and raising the embryos at a lower temperature, emergency granulopoiesis remained profoundly impaired (Fig. 3c). Similarly, direct overexpression of colony stimulating factor 3a (Csf3a/G-CSF), a haemopoietic growth factor relatively specific for neutrophils, resulted in vigorous neutrophil expansion in WT but failed to rescue the neutropenia of *mne* (Fig. 3d,e). Collectively, these data identify intact *mne* locus function as a genetic requirement for myeloid development that impacts the neutrophil lineage during both homeostatic and emergency granulopoiesis.

Since neutrophils and macrophages share a common progenitor, the requirement for Zbtb11 in macrophage development was also investigated. The temperature sensitivity of *mne* was used to examine the requirement of Zbtb11 for macrophage development across a range of phenotypic severities, with macrophages quantified at restrictive (32 °C), normal (28 °C) and permissive (23 °C) temperatures. In all cases, the population sizes of macrophages were not significantly different between *mne* and WT, showing that at 48 h.p.f. Zbtb11 deficiency does not impair basal macrophage development (Fig. 3f,g). In addition, macrophage replenishment following ablation was not dependent on intact *mne* locus function (Fig. 3h; Supplementary Fig. 4). At later time points (72–96 h.p.f.), macrophage numbers reduce in *mne* (Supplementary Fig. 4d), likely reflecting the failure to sustain definitive haemopoiesis. The data presented in Fig. 3, however, demonstrate that in primitive and early in definitive haemopoiesis, there is a much greater requirement for intact *mne* function in the neutrophil lineage than there is in the macrophage lineage.

### Zbtb11 is a direct target of major myeloid regulators

**Figure 4:**
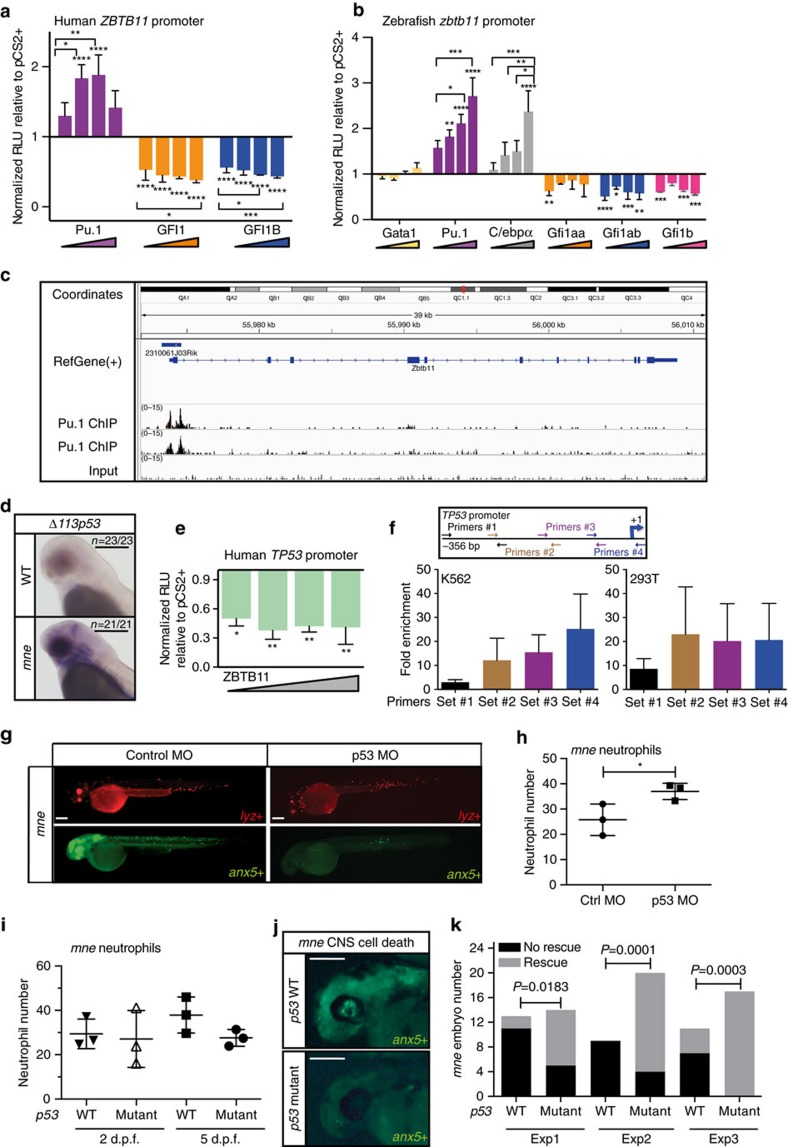
ZBTB11 is regulated by myeloid transcription factors and directly represses *TP53*. (**a**) Transient co-transfection of human ZBTB11 2.9 kb promoter luciferase reporter and transcription factors into 293T cells shows ZBTB11 is regulated by PU.1 (positively) and GFI1a/b (negatively). Triangles represent increasing concentration of transcription factors (*n*=3 experiments; mean ±s.e.m.; two-way ANOVA). (**b**) A zebrafish *zbtb11* 2.3 kb promoter reporter is positively regulated by Pu.1 and C/ebpα, and negatively regulated by all three Gfi1 paralogs. Triangles represent increasing concentration of transcription factors (*n*=3 experiments; mean ±s.e.m.; two-way ANOVA). (**c**) ChIPseq shows PU.1 occupies the *Zbtb11* locus in mouse granulocytes at the promoter, 5′ untranslated region of exon 1 and within intron 1. (**d**) Whole-mount *in situ* hybridization shows overexpression of *Δ113p53* in the brain at 48 h.p.f. in *mne* but not phenotypically WT sibling embryos. (**e**) Transient co-transfection of ZBTB11 and a human *TP53* luciferase reporter into 293T cells shows direct repression of *TP53* by ZBTB11. Triangle represents increasing concentration of ZBTB11 (*n*=3 experiments; mean ±s.e.m.; two-way ANOVA). (**f**) ZBTB11 is enriched at the *TP53* locus by ChIP–qPCR in human K562 (endogenous ZBTB11) and 293T HEK cells (overexpressed mouse ZBTB11). Using four primer sets tiled across the *TP53* promoter, primer set 1 yields little enrichment over normal rabbit serum control, while primer sets 2–4 show 12–25-fold enrichment (K562: *n*=5 experiments, mean ±s.e.m.; 293T: *n*=2 experiments, mean ±s.e.m.). (**g**) Antisense morpholino oligonucleotide knockdown of *tp53* suppresses excessive apoptosis and increases neutrophil number in *mne* embryos. (**h**) Quantification of *mne* neutrophils in control and *tp53* morphants (*n*=3 experiments; mean ±s.e.m.; two-tailed paired *t*-test). (**i**) Quantification of *mne* neutrophils in *tp53* WT and *tp53*^*M214K/M214K*^ at 2 and 5 d.p.f.; (*n*=3 experiments; two-tailed paired *t*-test). (**j**) Cell death marked by Annexin secA5-mVenus is prominent in *mne* CNS on *tp53* WT background and rescued on *mne/ tp53*^*M214K/M214K*^. (**k**) 2 × 2 Contingency table *χ*^2^ analysis shows rescue of CNS cell death in *mne* on the *tp53*^*M214K/M214K*^ mutant background. Data for three independent experiments; Exp1, *n*=13, 14; Exp2, *n*=9, 20; Exp3, *n*=11, 17; exact *P* values are shown. Where indicated: *P≤0.05; **P≤0.01; ***P≤0.001; ****P≤0.0001; scale bars, 300 μm (**d**), 200 μm (**g**,**j**).

Zbtb11-dependent transcriptional networks and its upstream genetic regulators have not been defined. To determine where Zbtb11 is placed with regard to the known haemopoietic transcriptional hierarchy, 2.9 kb of the human *ZBTB11* promoter and 2.3 kb of the zebrafish *zbtb11* promoter were cloned and assayed for activity in the presence and absence of increasing concentrations of different human or zebrafish haemopoietic transcription factors in 293 human embryonic kidney (HEK) cells. The myeloid specification determinant Pu.1 (ref. 21) positively regulated both zebrafish and human *ZBTB11* promoter reporters, whereas the erythroid transcription factor Gata1 did not (Fig. 4a,b), further supporting a myeloid-specific role for ZBTB11. Likewise, GFI1 and C/EBPα transcription factors, also implicated in myeloid specification^22^, respectively repressed and activated both zebrafish and human *ZBTB11* promoter reporters in a dose-dependent manner (Fig. 4a,b). These findings are consistent with published chromatin immunoprecipitation (ChIP) sequencing data examining genome-wide loci occupancy for a series of haemopoietic transcription factors including PU.1 and GFI1 in mouse HPC7 haemopoietic progenitor cells^23^, and functionally demonstrate regulation of the *ZBTB11* promoter specifically by these myeloid regulators. In addition, ChIP sequencing of mouse granulocyte chromatin demonstrated PU.1 occupancy at the *Zbtb11* locus (Fig. 4c) in granulocytes. In *mne* neutrophils, canonical *Pu.1 (Spi1b)* expression is slightly elevated compared to WT at 48 h.p.f. (logFC=0.57; FDR=0.043), which could indicate Pu.1 modulation by a Zbtb11-mediated potential negative feedback loop, though this remains to be explored. Collectively, these data identify a new myeloid transcription factor-ZBTB11 axis that is evolutionarily conserved in fish and mammals.

### TP53 is a direct ZBTB11 target

To understand gene regulatory networks directed by Zbtb11 both globally and specifically in neutrophils, WT and *mne* RNA were prepared from both whole embryos and FACS-purified *mpx-EGFP* or *lyz:dsRed*-expressing cells, and subjected to global RNA expression profiling. Tp53 was elevated in *mne* compared to WT in both analyses (global (microarray): logFC=3.5; neutrophils (RNA sequencing (RNAseq)): logFC=2.91, FDR=0.0000001). Tp53 was of particular interest because of the known requirement for its downregulation during the maturation of haemopoietic lineages^7^,^8^. The exquisite sensitivity and far-reaching consequences of TP53 activation are balanced by sophisticated multi-layered regulation requiring stabilization, activation and release from Mdm2-mediated targeting for degradation before invocation of TP53 transcriptional networks^24^,^25^. It was important to determine, therefore, if upregulation of *tp53* transcripts in *mne* was accompanied by corresponding functional protein. The high levels of the *Δ113Tp53* alternative transcript shown by whole-mount *in situ* hybridization (WISH) in *mne* indicate that the upregulation of *tp53* transcripts in *mne* results in stabilized activated Tp53 protein capable of transactivating its target genes, which include *Δ113Tp53* (ref. 26). The increase in Tp53 protein activity is localized strongly in the brain, particularly in the cerebellum, the eye and mandibular mesenchyme (Fig. 4d). Co-expression of human ZBTB11 significantly repressed a *TP53* promoter-luciferase reporter in human 293 cells, suggesting a direct interaction of ZBTB11 with the *TP53* promoter (Fig. 4e). ChIP of endogenous ZBTB11 in human erythroleukaemic K562 cells and of overexpressed mouse ZBTB11 in 293T HEK cells confirmed occupancy at the *TP53* promoter (Fig. 4f) further supporting a direct, active role for ZBTB11 in transcriptional regulation of *TP53*.

TP53 orchestrates genetic pathways signalling both cell death and cell cycle arrest. To understand if either or both of these mechanisms operate as a consequence of Zbtb11 deficiency, a Tg(*ubi:secA5-mVenus*) reporter, in which Annexin 5-mVenus expression serves as a marker for cell death, was crossed onto *mne*. Quantification of Annexin 5^+^ cells demonstrated a significant increase in global apoptosis in *mne* (Supplementary Fig. 5b). This was associated with a corresponding cell cycle arrest at 48 h.p.f. as measured by an almost complete absence of EdU incorporation in *mne* (Supplementary Fig. 5c,d). We hypothesized that if the Zbtb11/Tp53 interaction were functionally important in neutrophil development, Tp53 knockdown in *mne* would restore neutrophil numbers. Taking advantage of the extensive apoptotic cell death phenotype in *mne*, particularly in the central nervous system, the Tg(*lyz:dsRed;ubi:secA5-mVenus*) reporter was again employed. It also served as an internal control to monitor efficacy of the *tp53* translation blocking morpholino used to knock down Tp53 levels, which would be predicted to rescue any Tp53-dependent cell death phenotype. Indeed, the abnormally high global cell death in *mne* embryos was normalized to WT levels in *mne tp53* morphants (Fig. 4g). The number of neutrophils in *mne tp53* morphants was also greater than in control morphants (Fig. 4h). However, when this analysis was repeated in *mne* on the *tp53*^*M214K/M214K*^ DNA-binding mutant background, the number of neutrophils was not significantly different either at 2 or 5 d.p.f. regardless of *tp53* status (Fig. 4i). This suggests that mitigating the excessive over-expression of Tp53 by morpholino knockdown may be beneficial for neutrophil number in *mne*, however, complete removal of all transcription-dependent Tp53 functions is not. Differences in coincident *Annexin V* and *lyz* reporter expression were not detected between *mne* and WT, suggesting that apoptosis of neutrophils is not the major biological process underlying granulocyte deficiency at this time point (Supplementary Fig. 5a). As an independent measure of the biological relevance of the Zbtb11–Tp53 axis, rescue of CNS cell death was scored in *mne* on both *tp53* WT and *tp53*^*M214K/M214K*^ backgrounds (Fig. 4j). Across triplicate experiments, CNS cell death was significantly rescued on the *tp53*^*M214K/M214K*^ background (Fig. 4k), but not the hydrocephalus and associated craniofacial defects typical of the *mne* pleiotrophic phenotype. This suggests that the *mne* phenotype is not due solely to a stress-response induction of Tp53, rather Zbtb11 contributes by fine-tuning Tp53 during development. Together these data establish the ZBTB11–TP53 axis as a new, evolutionarily conserved pathway functionally contributing to normal neutrophil development.

### Zbtb11 Cys116 is key for HHCC domain and *TP53* repression

**Figure 5:**
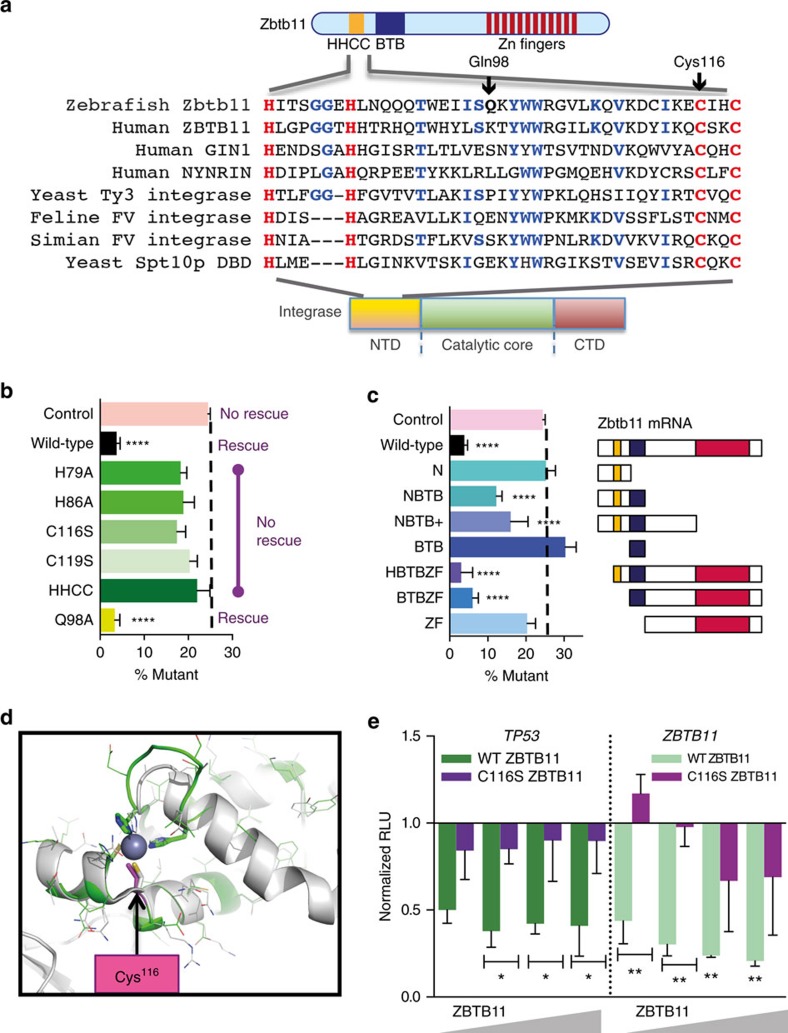
Cys116 is central in the HHCC domain and required for *TP53* repression. (**a**) A pair of conserved His and Cys residues (in red) in Zbtb11 align with those in the HHCC domain of integrase genes, and two human genes, *GIN1* and *NYNRIN*. Blue, conserved amino acids. (**b**) Mutation of each His or Cys residue (H79A, H86A, C116S, C119S) or all four (HHCC), but not mutation of a non-conserved *gln* (Q98A) abrogates Zbtb11 bioactivity in an *in vivo mne* rescue bioassay. (*n*≥3 experiments; mean ±s.e.m.; *χ*^2^ analysis; ****P≤0.0001). (**c**) Deletion of N terminus (HBTBZF, BTBZF) or zinc fingers (NBTB, NBTB+) does not abrogate rescue by Zbtb11. (*n*≥3 experiments; mean ±s.e.m.; *χ*^2^ analysis; ****P≤0.0001). Yellow box, HHCC domain; blue box, BTB domain; red box, zinc finger domain. (**d**) Modelling of amino acids 77–123 of zebrafish Zbtb11 using the integrase HHCC structure predicts a new domain in Zbtb11 that can form a zinc finger with each of the paired His and Cys residues, including Cys116 (cerise), coordinating a central metal ion (blue sphere). Green, homology model; grey, template. Conserved His and Cys residues are shown as thick sticks in the homology model. (**e**) Transient co-transfection of ZBTB11–C116S and a human *TP53* or *ZBTB11* luciferase reporter into 293T cells shows Cys116 is indispensible for the *TP53* and autoregulatory repressor function of ZBTB11 (*n*=3 experiments; mean ±s.e.m.; two-way ANOVA; *P≤0.05; **P≤0.01). The WT ZBTB11 data are identical to Fig. 4e because it was the contemporaneous control for this C116S data.

To unveil the biochemical mechanism underpinning the impact of the C116S mutation on Zbtb11 function, we investigated predicted structural motifs. Zbtb11 shares with other ZBTB family members a conserved BTB domain thought to be important for protein–protein interactions^27^ and 4 C-terminal zinc finger double domains overlapping 12 predicted Krüppel zinc fingers. Unusually among ZBTB proteins, Zbtb11 has an extended N-terminal domain with no recognized homology to predicted motifs or function, yet this contains the *mne* C116S mutation. Multiple sequence alignment of the region encompassing Cys116 revealed a paired His and Cys motif completely conserved across species (Fig. 5a). These amino acids are positioned similarly to those in the HHCC zinc finger in foamy virus integrase^28^,^29^ and identically to two human genes, GIN1 (gypsy retrotransposon integrase-like protein 1)^30^ and NYNRIN (NYN domain and retroviral integrase containing)^31^, forming a potential N-terminal HX_6_H(X_29_)CX_2_C zinc finger motif (Fig. 5a). The functional requirement for each of these conserved His/Cys residues was tested using a series of Zbtb11 point mutants in an *in vivo* bioassay based on *mne* rescue. Overexpression of Zbtb11 mRNA with mutation of any or all four of the His/Cys residues failed to rescue the *mne* phenotype (Fig. 5b,c). Wild-type Zbtb11 mRNAs with no mutation or mutation of a non-conserved Gln98 residue (Fig. 5a,b) both rescued *mne* function. Hence, each of the four residues of the HHCC motif is required for Zbtb11 function, supporting its functionality as a discrete motif. Furthermore, deletion mutants lacking the carboxyl-terminal zinc finger domains were able to rescue (Fig. 5c), consistent with a prior study demonstrating their dispensability for repression of the metallothionein promoter^19^. Of note, deletion of the N terminus did not interfere with functional rescue by Zbtb11 in this bioassay, suggesting that the steric consequences of point mutation of these four key residues are more detrimental to whole protein function than complete absence of this domain. To independently corroborate this observation about the residual functionality of N-terminally deleted Zbtb11, cell cycle progression was examined. Cell cycle progression, demonstrated by EdU incorporation, presents an almost categorical phenotypic difference between WT and *mne*, being present and absent, respectively. Overexpression of N-terminally deleted Zbtb11 but not the C116S mutant Zbtb11 again rescued *mne* gross morphology and concomitantly restored cell cycling activity (Supplementary Fig. 6).

To gain further insight into the impact of the C116S *mne* mutation on Zbtb11 structure, three foamy virus integrase structures complexed with manganese and containing an HHCC motif similar to Zbtb11 (Fig. 5a) were used as a template for homology modelling of zebrafish Zbtb11 amino acids 77–123. This demonstrated a zinc finger structure in which each of the conserved His/Cys residues including Cys116, coordinates the metal ion (Fig. 5d).

To functionally examine the direct consequence of the T→A (C116S) *mne* mutation on the ZBTB11-*TP53* promoter interaction, ZBTB11 was engineered to contain the C116S mutation and co-transfected with a *TP53* promoter-driven reporter. Compared to WT ZBTB11, the mutant failed to show significant repression of *TP53* at any of the doses measured (Fig. 5e). Furthermore, whereas WT ZBTB11 was found to regulate its own promoter, mutation of C116S resulted in failure of this autorepression (Fig. 5e). Together these data indicate that Cys116 is a critical component of a novel zinc finger structure within the N-terminal domain of ZBTB11 whose integrity is required for its activity as a transcriptional repressor of its target, *TP53*.

## Discussion

We have identified a role for the previously enigmatic Zbtb11 protein in myeloid development. The Zbtb11 requirement for granulopoiesis is already apparent during primitive haemopoiesis reflected by the paucity of neutrophils in *mne* compared to WT at 48 h.p.f. and this requirement continues into definitive haemopoiesis where haemopoietic stem cell-dependent neutrophil expansion fails to occur in *mne* in response to immune challenge or cytokine stimulation. Thus Zbtb11 appears to be important for both basal and emergency granulopoiesis. That macrophage number is indistinguishable between WT and *mne* at 48 h.p.f. and that *mne* macrophages can reconstitute after ablation indicate that intact Zbtb11 is dispensable for primitive macrophage development but becomes rate-limiting as reliance on HSC self-renewal and differentiation increases and becomes absolute following the onset of definitive haemopoiesis. The differential timing between neutrophil and macrophage depletion suggests Zbtb11 has an essential role in establishing a full complement of neutrophils, whereas with macrophage and other lineages, the later depletion is reflective of a broad failure of myelopoiesis. The positioning of ZBTB11 downstream of the master myeloid regulators PU.1, C/EBPα and GFI1 in the haemopoietic transcriptional hierarchy suggests ZBTB11 may potentially act at the level of progenitors to direct proliferation, differentiation and/or survival towards amplifying neutrophil number. This is supported by the data showing that Zbtb11 dysfunction results not only in a quantitative defect in neutrophils but also a qualitative defect manifest in the high proportion of immature neutrophil lineage cells in *mne*.

Although we have clearly positioned *ZBTB11* within the haemopoietic transcription factor hierarchy, its precise mechanism of action remains elusive. Pathway analysis generated from RNAseq data from *mne* versus WT neutrophils reveals central roles for Zbtb11 in RNA processing, DNA replication and repair as well as cell death and survival, and we have presented functional data validating roles in both DNA replication and cell death, where Zbtb11 deficiency results in markedly increased apoptosis and virtually absent DNA synthesis. Studies describing a role for TP53 in haematopoiesis, specifically the granulocytopenia accompanying overexpression of Trp53 in the Mdm2 knockout mouse^9^, prompted us to study whether the upregulation of *tp53* in *mne* was in response to cell stress or whether it was due to derepression in the absence of fully functional Zbtb11. The biochemical evidence shows that not only does ZBTB11 repress *TP53* but that it requires Cys116 for this function, suggesting that the high levels of Tp53 in *mne* may at least in part be due to derepression of *tp53* by mutant Zbtb11. Van Nostrand *et al*.^32^ showed that ectopic expression of Trp53 during development results in a pleiotrophic phenotype similar to that seen in the CHARGE syndrome in humans, including craniofacial, cardiac and eye defects. Many aspects of this phenotype are mirrored in *mne*, and may partially explain the pleiotropism that results from Zbtb11 dysfunction and high levels of Tp53 during development in *mne*.

Knockdown of *tp53* overexpression in *mne* by a widely used and extensively characterized translation-blocking morpholino partially rescued neutrophil number, pointing to a novel Pu.1–Zbtb11–Tp53 pathway for regulation of neutrophil development. This observation was in contrast to the *tp53* mutant data, which demonstrated that in the context of a *tp53*^*M214K/M214K*^ allele encoding transcriptionally dead Tp53 protein, neutrophil number was not normalized in *mne*. Discrepancies between morpholino knockdown and stable genetic mutant phenotypes have been highlighted recently^33^ but do not necessarily mean that either outcome is incorrect^34^. Indeed, accurate interpretation of TP53 data has been notoriously challenging. What could these two observations mean? It is possible that morpholino knockdown corrects the overly high levels of Tp53 in *mne* to a subtle level that allows for amelioration of the neutrophil deficit, while genetic inactivation of *tp53* removes the normal level of control achieved by low levels of Tp53 and presents a different regulatory landscape that prevents rescue of the neutrophil deficiency. It is also possible that compensatory mechanisms by TP53 family members p63 and p73 may come into play in the *tp53* mutant^35^,^36^,^37^. It is well known that TP53 is increased in response to various types of cell stress including nucleolar stress, which affects erythropoietic output in Diamond Blackfan Anemia^38^,^39^,^40^,^41^. Could the *mne* phenotype be attributable solely to stress response overexpression of Tp53? Neither the genetic inactivation nor morpholino knockdown data can rescue *mne* gross morphological defects, which strongly supports the notion that the phenotype observed in *mne* is not due solely to activation of Tp53 stress response pathways and that Zbtb11 can exert its biological effects through a Tp53-independent mechanism. With regard to CNS cell death, the morpholino and genetic data are corroborative, demonstrating that CNS cell death is dependent on both an intact *mne* locus and functional tp53. This is consistent with an additional role for the ZBTB11–TP53 pathway outside of haemopoietic development.

We sought to investigate whether the requirement for *zbtb11* during zebrafish myeloid development was cell-autonomous by transient overexpression approaches. However, we were not able to confirm reliable, reproducible expression of Zbtb11–GFP targeted to myeloid cells from transient, mosaic expression, even using a Gal4/UAS approach in an attempt to amplify the signal. Future endeavours to address this issue experimentally will require stable transgenic approaches optimized for Zbtb11 reporter expression in zebrafish, or approaches in other animal models.

The non-catalytic HHCC domain that resides in the N-terminal domain of retrovirus and related retrotransposon integrases, such as HIV-1 (human immunodeficiency virus 1), is crucial for determining the conformation and therefore activity of the integrase and infectivity of the virus^42^. The tetrahedral coordination of a single zinc ion by the His and Cys residues in this domain stabilizes the integrase allowing multimerization and more effective catalytic activity. The canonical zinc binding motif is HX_3–7_H(X_23–32_)CX_2_C (ref. 43). Studies of HIV-1 integrase have revealed two distinct interconverting D- and E-conformations that are determined as a result of how the HHCC domain specifically coordinates a Zn^2+^ ion, underscoring the importance of the HHCC domain for overall function of integrase^44^,^45^. The surprising identification of a new HHCC (HX_6_H(X_29_)CX_2_C) zinc finger in the N-terminal domain of Zbtb11 that underpins its function as a transcriptional repressor provides the first functional data for the HHCC domain in a human protein. The evolutionary conservation of the HHCC motif across >22 vertebrate species of Zbtb11 further highlights its importance for function. The identification of a cellular function for the HHCC domain in Zbtb11 supports the previously untested hypothesis^31^ that the homologous uncharacterized HHCC domain in NYNRIN and GIN1 also performs a cellular function. The different possibilities for HHCC tetrahedral coordination of the Zn^2+^ ion identified in HIV-1 integrase and the functional consequences for enzyme activity, suggest a complex novel regulatory role mediated through this domain. This complexity is reflected in the Zbtb11 *in vivo* bioassay data, where the absence of the HHCC domain allows Zbtb11 to rescue the *mne* phenotype but mutation of any of the four metal ion-coordinating amino acids does not. The notion that steric hindrance by an incorrectly folded N terminus is potentially more detrimental than its complete absence has previously been documented^46^, and in the case of zinc finger proteins is supported by evidence showing that metal coordination participates early in the folding process and is critical for proper folding of these proteins^47^,^48^. We propose that misfolding of the N terminus incurs a more severe penalty on Zbtb11 protein folding integrity and stability than complete absence of the N terminus, which may still allow for correct modular folding of the remaining protein domains. However, this question will only be resolved by biophysical data. Since removal of the C-terminal zinc fingers remarkably does not appear to impede function, either in our *in vivo* bioassay or a single previous *in vitro* study^19^, the newly identified HHCC zinc finger could serve to preserve Zbtb11 function in this context. It is thought that in viruses this zinc finger recognizes viral DNA^42^ and we have shown a requirement for the intact HHCC domain for recognition of cellular DNA through the transrepression and autorepression functions of Zbtb11. It remains an intriguing possibility that the cellular HHCC domain may also recognize viral DNA, potentially as part of the host immune response.

Zbtb11 is a previously under-studied protein to which we now ascribe a biological function squarely positioning it within the haemopoietic transcription factor hierarchy as a regulator of basal neutrophil development and emergency granulopoiesis. In addition, we have identified a genetic and biochemical pathway connecting ZBTB11 and TP53 that now merits consideration in all tissues in which both Tp53 and Zbtb11 are expressed. Lastly, we have identified a novel integrase-like HHCC domain in Zbtb11. To our knowledge, we have provided the first cellular function for this domain in a human protein, specifically the transcriptional repressor activity of Zbtb11, with potential functional implications for regulatory domains of other human proteins. Together, these studies provide a basis for understanding how Zbtb11 dysregulation may contribute to disease pathogenesis and opens a new window on virally derived cellular domain function.

## Methods

### Animals

Strains: St Kilda Wild Type (SKWT; local pet shop, St Kilda, Victoria, Australia), WIK, AB, AB* (Zebrafish International Research Centre, Eugene, Oregon, USA) and Tübingen (Max-Planck-Institut für Entwicklungsbiologie, Tübingen, Germany). *Marsanne* (*mne*^gl11^) is a novel mutant isolated from an ethylnitrosourea (ENU) mutagenesis screen^49^. Primary transgenic lines were as follows: Tg(*mpx:EGFP*)^i114^ (ref. 50), Tg(*lyz:dsRed*)^nz50Tg^ (ref. 51), Tg(*gata1a:dsRed*)^sd2Tg^ (ref. 52), Tg(*ubi:secAnnexinV-mVenus)*^mq8Tg^ (ref. 53), Tg(*mpeg1:Gal4FF*)^gl26^ (ref. 54) and Tg(*UAS:nfsb-mCherry*)^c264^ (Zebrafish International Stock Center, Eugene, OR). Compound and mutant lines were generated by intercrossing. Fish were housed in the Ludwig Institute for Cancer Research Aquarium and ARMI FishCore, and mice were housed in the WEHI mouse facility using standard husbandry practices. Experiments were performed according to protocols approved by the Animal Ethics Committees of the Ludwig Institute for Cancer Research, The Walter and Eliza Hall Institute of Medical Research and Monash University.

### Genotyping

From 48 h.p.f., *mne* embryos were readily recognized in a Mendelian proportion by their pleiotropic phenotypes including small dark eyes, neural opacity, enlarged fourth ventricle and neutropenia. Younger *mne* embryos were genotyped by PCR–RFLP (restriction fragment length polymorphism) using exon 2 primers (oligonucleotides, Supplementary Table 1) in 20 μl reactions with Phusion polymerase (New England Biolabs, MA) and supplied GC buffer; 95 °C, 2 min followed by 45 cycles at 95, 60 and 72 °C for 30, 30 and 60 s, respectively, and 1 cycle of 10 min at 72 °C. PCR products were digested with Nsi1, which digests only the WT allele since the T→A *mne* allele abolishes this site, and digestion products resolved alongside corresponding uncut sample by agarose gel electrophoresis.

### Positional cloning

Positional cloning was initiated by a genome scan on embryos from an F2 generation WIK pedigree mapping pair, MX95 (ref. 55). Two independent pools of 40 WT and 40 mutant embryos were scored against a panel of simple sequence length polymorphism markers selected to give ∼10 cM coverage across all chromosomes. Bulk segregant analysis placed *mne* on chromosome 6. Genomic regions potentially closer to the mutant locus than the closest linked simple sequence length polymorphism markers were identified and primers designed to amplify ∼1–1.5 kb products by PCR from individual mapping pairs. Direct sequencing of the PCR products allowed detection of single-nucleotide polymorphisms in these regions. Single-nucleotide polymorphisms that generated useful RFLPs were selected for scoring, and individual mutant embryos recombinant at more distant markers were scored at these RFLPs. This narrowed the genetic interval to a 50 kb region containing a single gene, *zbtb11*, which was sequenced to identify the mutation underpinning *mne*. Supplementary Table 1 lists oligonucleotide sequences used.

### FACS sorting and RT–qPCR

Haemopoietic populations were obtained from adult zebrafish whole kidney marrow from Tg(*gata1-dsRed*) transgenic animals. Erythrocytes were sorted first on the basis of *gata1*-dsRed fluorescence and remaining populations were sorted on the basis of their physical characteristics (forward and side scatter). Triplicate sample replicates obtained from single complementary DNAs were subjected to quantitative PCR using primers in Supplementary Table 1. Results were normalized using the ΔΔCt method, and compared to expression in whole kidney marrow. The purity of the myeloid and erythroid populations was directly confirmed by concurrent qPCR for expression of *gata1* and *spi1*, which demonstrated low/absent expression of *gata1* in the myeloid population and low/absent levels of *spi1* in the erythroid population (Supplementary Fig. 7).

### Cloning

Primers for cloning are shown in Supplementary Table 1. In brief, 48 h.p.f. *mne* cDNA was used for PCR amplification of zebrafish *zbtb11* cDNA. The PCR product was cloned into pBluescript II SK+ (Stratagene) then directionally cloned into pCS2+ ClaI/XhoI sites to generate *mne*-*zbtb11*-pCS2^+^. Mutagenesis was used to generate WT-*zbtb11*-pCS2^+^ from the *mne* *zbtb11* clone. Human *ZBTB11* was amplified from K562 cells and mouse *Zbtb11* was amplified from mouse thymocyte cDNA (a kind gift from Dr Matthew McCormack). Human (2.9 kb) and zebrafish (2.3 kb) *Zbtb11* promoters were cloned from K562 and zebrafish genomic DNA, using In-Fusion (Clontech) into pGL3-luc (Promega). All constructs were verified by nucleotide sequencing.

### Microinjections

Deletion and point mutation Zbtb11 constructs as carboxy-terminal GFP fusions were synthesized by inverse PCR or mutagenesis (QuickChange Lightning, Stratagene or Q5 mutagenesis, NEB) and sequence verified (for primers see Supplementary Table 1). Capped mRNA for microinjection was synthesized from Not 1 linearized template using SP6 mMESSAGE mMACHINE (Ambion). Fertilized 1- to 2-cell embryos were microinjected with 1–2 nl synthetic mRNA, Zbtb11 ATG morpholino oligonucleotide (MO), *tp53* MO or control MO (250 μM in H_2_O; Gene Tools, Philomath, OR; see Supplementary Table 1 for sequence) traced where appropriate by mixing 1:1 with 5% rhodamine–dextran (in 0.2 M KCl).

### *In vivo* bioassay

For the *in vivo* bioassay embryos from a *mne*^+/−^ incross were injected with WT (rescue control) or test Zbtb11 mRNA and seeded at ∼40 embryos/dish. To determine rescue, gross morphological phenotype was scored at 48 h.p.f. for all embryos and the percentage of mutant versus WT embryos was determined for each Zbtb11 construct and compared against the non-injected controls (Mendelian ratio of ∼25% mutants).

### CRISPR/Cas alleles

Oligonucleotides for guide RNA (sgRNA) synthesis were designed using CHOP–CHOP (sequence in Supplementary Table 1). A *zbtb11*-specific oligonucleotide containing a T7 polymerase recognition sequence at the 5′ end was annealed to the constant oligonucleotide (encoding the reverse complement of the tracrRNA tail) via an overlapping homologous region^56^. Nucleotides were filled in by T4 DNA polymerase to create a double-stranded template for sgRNA synthesis using T7 Polymerase. RNA integrity was monitored by gel electrophoresis. Cas9 protein (New England Biolabs) complexed with sgRNA was injected into either Tg(*mpx:EGFP*) or Tg(*lyz:dsRed*) embryos. Injected embryos were screened genotypically by sequencing and phenotypically for the *mne* phenotype. F0 embryos were raised and out-crossed onto *mne* to determine founders containing non-complementing CRISPR alleles. Indel mutations were characterized in the F1 generation.

### Microarray

Total RNA was isolated from three biologically independent pools of WT and *marsanne* embryos using Trizol/chloroform extraction and isopropanol precipitation and treated with RNase-free DNase (Ambion). Any remaining phenol was removed using an RNeasy micro kit (Qiagen). Samples were provided to the Ramaciotti Centre for Genomics (University of New South Wales, Australia) for QC and processing. Input was 100 ng of total RNA, samples were processed using the Affymetrix WT Plus kit with no amplification and hybridization was to Affymetrix Zebrafish Gene Array 1.0 ST (Affymetrix). Data were analysed using Bioconductor (Bioconductor—Open Source Software for Bioinformatics ( http://www.bioconductor.org) Copyright 2017) and R version 3.2.5 (The R Project for Statistical Computing ( https://www.r-project.org/)) packages. Expression values for all genes were calculated using the robust multi-array average method^57^. Data for biological replicates clustered into their separate groups corresponding to WT and *marsanne*. For the identification of genes with differential expression between groups, fold-change cutoff (≥2.0) and *P* value cutoff (≤0.05) were used for differential expression.

### WISH and *O*-dianisidine staining

WISH was performed using standard techniques^58^. Staining of haemoglobin by *O*-dianisidine was performed for 15 min at RT in 0.6 mg ml^−1^ *O*-dianisidine, 0.01 M sodium acetate (pH 4.5), 0.65% H_2_O_2_ and 40% vol/vol ethanol. Embryos were imaged on an Olympus MVX10 microscope and processed in Fiji^59^, where head and tail images of the same embryo were spliced to maintain in-focus focal plane, a dashed line indicates the junction.

### EdU labelling

Embryos were phenotyped at 48 h.p.f. and scored prior to labelling. EdU incorporation was achieved by soaking embryos in 0.2 mM EdU (5-ethynyl-2′-deoxyuridine; Invitrogen), 10% DMSO in E3 medium on ice for 60 min. Following washing (3 × 5 min in E3) and fixation in 4% PFA/PBS for 90 min, embryos were again washed (3 × 5 min in PBST; 1 × dH_2_O) and incubated in acetone at −20 °C for 7 min, then permeabilized in 1% DMSO/1% Triton-X100/PBS for 1 h at RT. EdU incorporation was detected by incubation in PBS/0.3% Triton-X100 containing 0.2 mM Alexafluor-555 (Life Technologies), 0.1 M L-ascorbic acid, 100 mM Tris pH 8.5, and 2 mM CuSO_4_ for 2 h at RT and washed (5 × 5 min PBST) prior to imaging. Embryos were imaged on an Olympus MVX10 fluorescence microscope and the number of EdU positive cells in caudal haemopoietic tissue counted in Fiji using the Find Maxima algorithm.

### *In vivo* neutrophil and macrophage studies

Stimulation of granulopoiesis was with microinjection of 0.1 and 0.05 ng csf3a mRNA or intravascular injection of freeze-killed *Talaromyces* (formerly *Penicillium*) *marneffei* at 48 h.p.f. Inducible macrophage ablation using Tg(*mpeg1:Gal4FF/UAS:nfsb-mCherry/mpx:EGFP*) embryos generated by inter-crossing was achieved with 10 mM metronidazole (Sigma M3761) treatment beginning at 36 h.p.f. and continuing for 11 h. At 48 h.p.f., embryos were placed in fresh E3 medium and imaged (*t*=0 h post treatment (h.p.t.)) to enumerate macrophages and neutrophils, then incubated a further 53 h.p.f. (*t*=53 h.p.t.) and again imaged. Leukocyte numbers in the caudal haemopoietic tissue region (posterior to end of yolk extension) were quantified either by manual counting or by leukocyte units^60^.

### Chromatin immunoprecipitation–quantitative PCR

ChIP of endogenous ZBTB11 from K562 cells was performed on 1 × 10^7^ cells grown to log phase and cross-linked with 1% formaldehyde for 10 min at RT then quenched in 0.125 M glycine. Sonication of the crude nuclear fraction was conducted for 10 rounds of 30 s on/30 s off using Bioruptor (Diagenode) to achieve chromatin fragmentation of ∼550 bp. A polyclonal anti-ZBTB11 antibody validated for immunoprecipitation (#A303-240A; Bethyl Laboratories) and Protein A Dynabeads were used for chromatin immunoprecipitation. This antibody was also validated in-house by immunoprecipitation of overexpressed human ZBTB11–GFP fusion proteins followed by detection with anti-GFP antibody (Roche) on immunoblot. Following reverse cross-linking overnight at 65 °C, DNA was isolated and a PCR mix containing SYBR green (Roche) was prepared according to the manufacturer's specifications using primer sets tiled across the *TP53* promoter and negative control primer sets (Supplementary Table 1), and 1 μl diluted template DNA. qPCR was conducted on a 7500 Real-Time PCR machine (Applied Biosystems). Overexpression of mouse ZBTB11 in 293T HEK cells and ChIP with a monoclonal anti-mouse ZBTB11 antibody (WEHI) gave concordant enrichment of ZBTB11 at the *TP53* locus. Antibody was prepared by immunization of rats with mouse ZBTB11 KLH-conjugated SSEESYRAILRYLTNERC peptide. ELISA positive supernatants were tested by immunoblot for reactivity with mouse ZBTB11 and cross-reactivity with human or zebrafish Zbtb11 (negative for both). The selected clone (IC2) was validated by immunoprecipitation of overexpressed mouse ZBTB11–GFP fusion protein (Supplementary Fig. 8). Ct values were obtained and fold enrichment calculated taking into account primer amplification efficiency (AE) using the ΔΔCt method: fold enrichment=AE−(ΔΔCt), where ΔΔCt=(Ct(TP53)−Ct(Input))−(Ct(NRS)−C(Input)).

### Western blot

Fifty micrograms of cell lysate was electrophoresed on a denaturing reducing 4–12% polyacrylamide gel. Protein was transferred to Immobilon-FL membrane (Millipore), blocked for 2 h at RT in Odyssey blocking buffer (LI-COR) prior to incubation with ab84058 (abcam) at 1:500 dilution overnight at 4 °C. Signal was detected by anti-rabbit IRDye-680LT secondary antibody (LI-COR) on an Odyssey infrared detection system (LI-COR). See Supplementary Fig. 9 for uncropped western blot.

### RNA sequencing

At 48 h.p.f., single-cell suspensions were prepared from either WT embryos or from phenotype-sorted *mne*, and neutrophils FACS sorted into RNALater (Qiagen) on the basis of bright *mpx:EGFP* or *lyz:dsRed* fluorescence (FlowCore, Monash University, Victoria, Australia). To confirm correct gating, sample FACS-sorted populations were analysed under fluorescence microscopy and found to contain only fluorescent neutrophils. RNA was extracted from sorted cells using RNeasy Micro (Qiagen) and provided to the MHTP Medical Genomics facility (Monash Health Translational Precinct, Clayton, Australia) for QC, library preparation and sequencing. Details in brief: unstranded barcoded libraries were prepared using total RNA and Nugen Ovation RNA-Seq system V2 for amplification and cDNA generation, followed by Ovation Ultralow System V2 for library preparation. Hundred base pair paired-end sequencing was performed on Illumina HiSeq2 generating ∼20 M reads per sample. Data were QC'd using FastQC, ends trimmed using Trimgalore and sequence aligned to the zebrafish genome (GRCz10) using STAR. Counts were derived using HTseq-count and differentially expressed genes determined using limma+voom with a FDR=0.01 and logFC cutoff of 2.0 using Degust v0.21 (David R. Powell, Victorian Bioinformatics Consortium, Australia).

### ChIP sequencing

FACS was used to isolate Ly6G^+^CD11b^+^ granulocytes from the bone marrow of C57BL/6 mice. ChIP samples were prepared according to the standard Millipore/Upstate protocol and using the polyclonal anti-Pu.1 IgG (T-21 X: sc-352 X) from Santa Cruz. Libraries were prepared and sequenced using the Illumina TruSeq workflow. Reads were aligned to the mm10 build of the *Mus musculus* genome using Subread aligner^61^.

### Protein homology modelling

Zbtb11 amino acids 1–200 were used in a blastP homology search against the Protein Data Bank (PDB) resulting in 5/14 hits with Cys at C116 position which aligned with Foamy virus intasome protein. Three structures (PDB IDs: 3OYM, 3OYI, 3OYK) complexed with manganese were used as a template for homology modelling of Zbtb11 amino acids 77–123. Alignment was performed with Clustal version 2.0.9 (ref. 62) and modelling with Modeller version 9.12 (ref. 63).

### Luciferase assays

Promoter and increasing doses of transcription factor (10–100 ng) were co-transfected using Fugene 6 (Roche) into 293T HEK cells, maintaining the total amount of DNA constant. Transcription factors were all subcloned into the same vector (pCS2^+^) and activity normalized against vector alone. They were as follows: zebrafish: *gata1* and *scl* (ref. 64), *erg1* (ref. 65), *Pu.1* (ref. 58), *c/ebpα* (ref. 66), and *gfi1aa*, *ab* and *bb* (ref. 67); mammalian transcription factors: MSCV-PU.1-IRES-GFP, pENTR-GFI1 and pENTR-GFI1B (ref. 68), and *TP53* promoter pGL2-356bp (ref. 69). Dual luciferase assays were performed as per manufacturer instructions (Promega) and analysed on a CLARIOStar (BMG Labtech). Cell lines used in these studies were a kind gift of Professor Stephen M Jane and were routinely monitored, and confirmed negative for mycoplasma.

### Statistics

Group sizes were planned to be >10 embryos/genotype/experiment. In practice, the number of embryos was often much greater and limited by the maximum practical number of randomly selected embryos that could be analysed. If group sizes were smaller, it was due to limited embryo availability or loss during the experiment, and these were assumed to be randomly distributed across groups unless otherwise stated. Where appropriate to the hypothesis being tested, embryos were assigned as mutant or wild-type, either by phenotype and/or *post hoc* molecular genotyping. Otherwise, embryos were randomly assigned to experimental groups. For embryos <48 h.p.f., experiments were always blinded to genotype and hence scored blind, with genotype determined and allocated post data analysis. Descriptive and analytical statistics were prepared in Prism 6 (GraphPad Software Inc). *F*-tests were used to determine variance, which was always similar for parametric tests presented. Where variance was significantly different between groups, non-parametric tests were used. Unless otherwise stated, data are from ≥3 experiments: (1) for luciferase assays: mean ±s.e.m.; two-way ANOVA; Dunnett's multiple comparisons to determine significance of transcription factor versus vector alone and Tukey's multiple comparisons to determine significance between doses of a given transcription factor; (2) for *in vivo* leukocyte studies: mean ±s.e.m., two-tailed *t*-test; (3) rescue experiments: *χ*^2^ analysis. NS, *P*>0.05; *P≤0.05; **P≤0.01; ***P≤0.001; ****P≤0.0001.

### Data availability

The microarray data sets generated during the current study are available in the Gene Expression Omnibus repository, accession number GSE94532. The authors declare that all remaining data supporting the findings of this study are available within the article and its Supplementary Information Files or from the corresponding author upon reasonable request.

## Additional information

**How to cite this article:** Keightley, M.-C. *et al*. The Pu.1 target gene Zbtb11 regulates neutrophil development through its integrase-like HHCC zinc finger. *Nat. Commun.* **8,** 14911 doi: 10.1038/ncomms14911 (2017).

**Publisher's note:** Springer Nature remains neutral with regard to jurisdictional claims in published maps and institutional affiliations.

## Supplementary Material

Supplementary Information Supplementary Figures, Supplementary Tables.
