# Supplementary material for: The Pu.1 target gene Zbtb11 regulates neutrophil development through its integrase-like HHCC zinc finger
Source: Nat Commun. 2017 Apr 6;8:14911. doi: 10.1038/ncomms14911 (PMC5384227; doi:10.1038/ncomms14911)
Supplement: Supplementary Information — Supplementary Figures, Supplementary Tables. [file ncomms14911-s1.pdf]

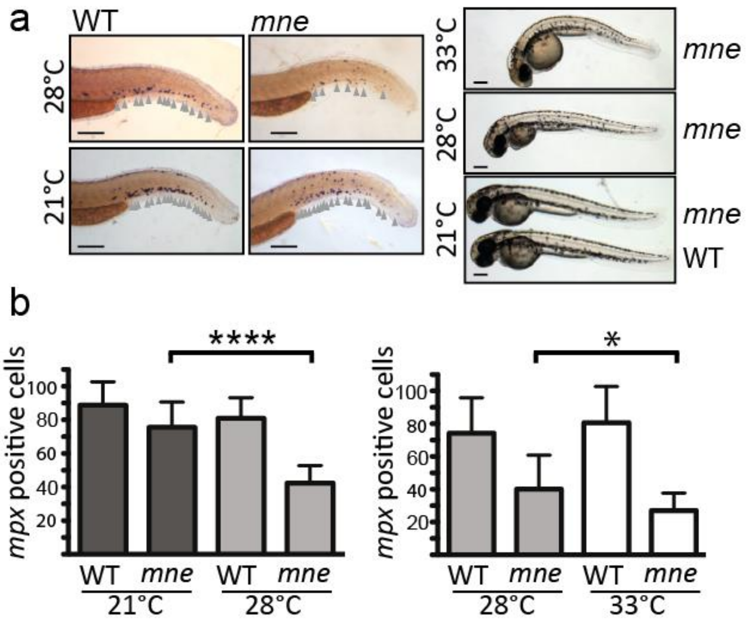

Supplementary Figure 1

Marsanne **is a temperature-sensitive** ***zbtb11*** **mutant (supports Fig. 1b)**

- **a.** Whole-mount *in situ* hybridization showing staining of *mpx*+ neutrophils and gross morphology demonstrates severity of the *mne* myeloid-failure phenotype and the overall dysmorphic phenotype of *mne* embryos varies with temperature. Grey arrows indicate neutrophils; scale bar = 200 µm;
- **b.** At lower temperature (21°C) neutrophil numbers are partly restored towards normal and at higher temperatures (33°C) the neutrophil deficiency is exacerbated. Left: *n*=14-16/group; Right: *n*=12-17/group; mean ±SD, two-tailed unpaired t-test.

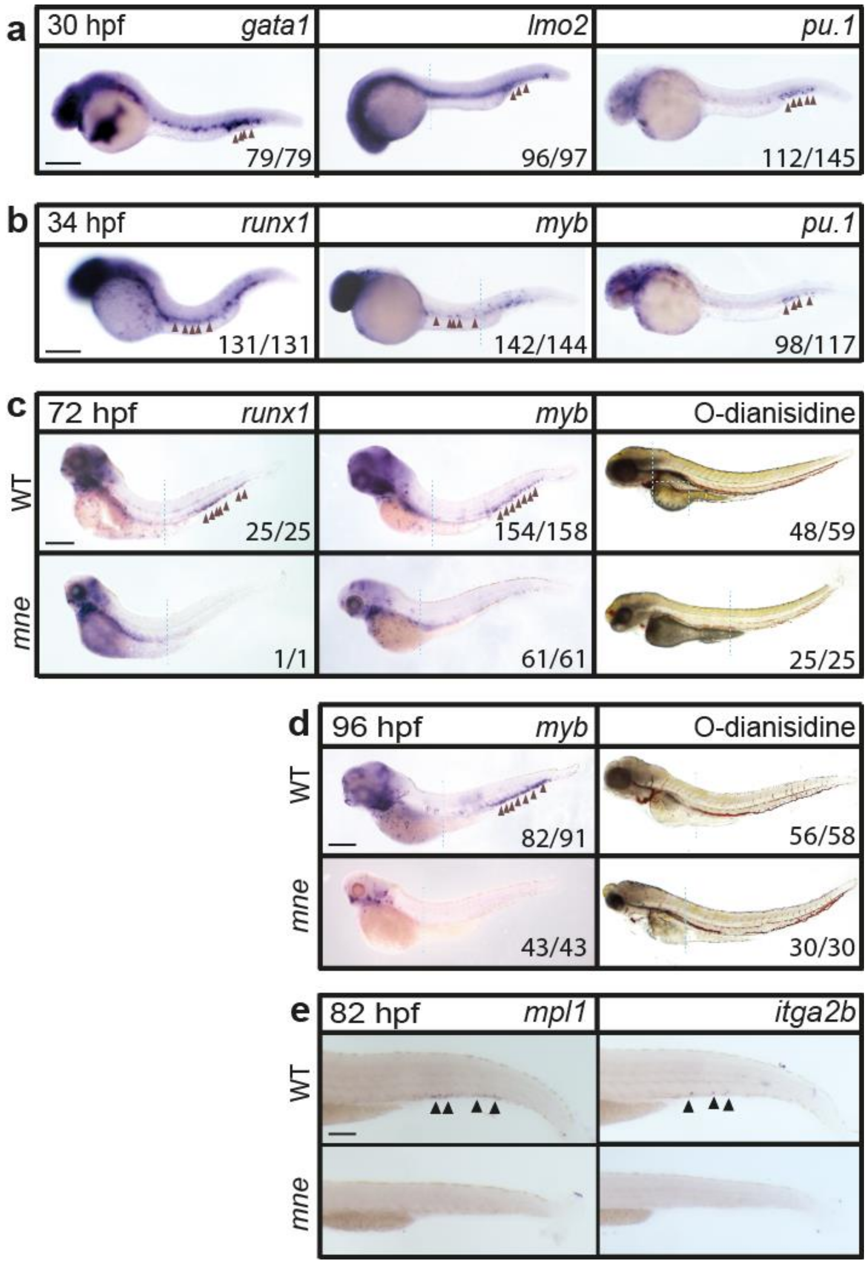

Supplementary Figure 2

Depletion of definitive haemopoiesis in *mne*
(supports Fig. 1b)

Whole-mount *in situ* hybridization showing:

- **a.** Erythromyeloid specification marked by *gata1*, *lmo2* and *pu.1* expression proceeds in *mne;*
- **b.** HSC specification marked by *runx1* and *myb* proceeds in *mne*.
- **c,d.** At 72 and 96 hpf, HSCs are depleted in *mne* while O-dianisidine staining shows haemoglobinised erythrocyte abundance is unaffected;
- **e.** Decreased numbers of cells expressing *mpl* and *itga2b* shows that thrombocytes are depleted in *mne.*

Where head and tail images of the same embryo were spliced to maintain in-focus focal plane, a dashed line indicates the junction; panels **a-e** scale bar = 200 μm.

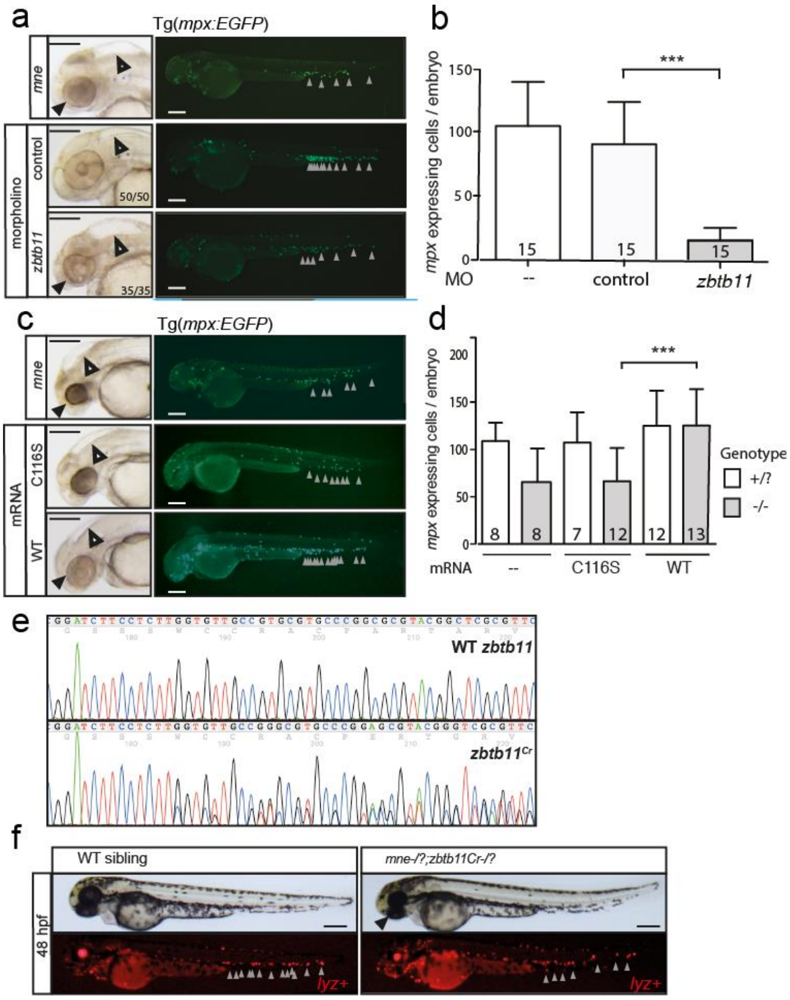

Supplementary Figure 3

Genetic proofs validate identification of Zbtb11 from positional cloning (supports Fig. 1d)

- **a.** Phenocopy of *mne* hydrocephalus phenotype (open arrow) and microphthalmia (solid arrow) phenotypes and neutrophil-depletion phenotypes (fluorescence images) by antisense morpholino oligonucleotide knockdown of Zbtb11 in WT embryos;
- **b.** Enumeration of neutrophils in morphant embryos represented in **a;** *n* numbers are shown within columns;
- **c.** Rescue of *mne* hydrocephalus (open arrow) and microphthalmia (solid arrow) phenotypes and neutrophil-depletion phenotype (grey arrows in fluorographs) by overexpression of WT but not C116S Zbtb11 mRNA in *mne* embryos;
- **d.** Enumeration of neutrophils in morphant embryos represented in **c**; *n* numbers are shown within columns;
- **e.** Additional *zbtb11* allele generated by CRISPR/Cas9 locus genome editing. Shown is sequence for WT *zbtb11* and a *zbtb11*^{Cr} CRISPR allele with predicted premature stop codon at amino acid 56;
- **f.** Non-complementation of *mne* allele by *zbtb11*^{Cr} allele in *mne;zbtb11*^{Cr} transheterozygotes (black arrow, microphthalmia; grey arrows, neutrophils); Panels **a, c, f** scale bar = 200 μm.

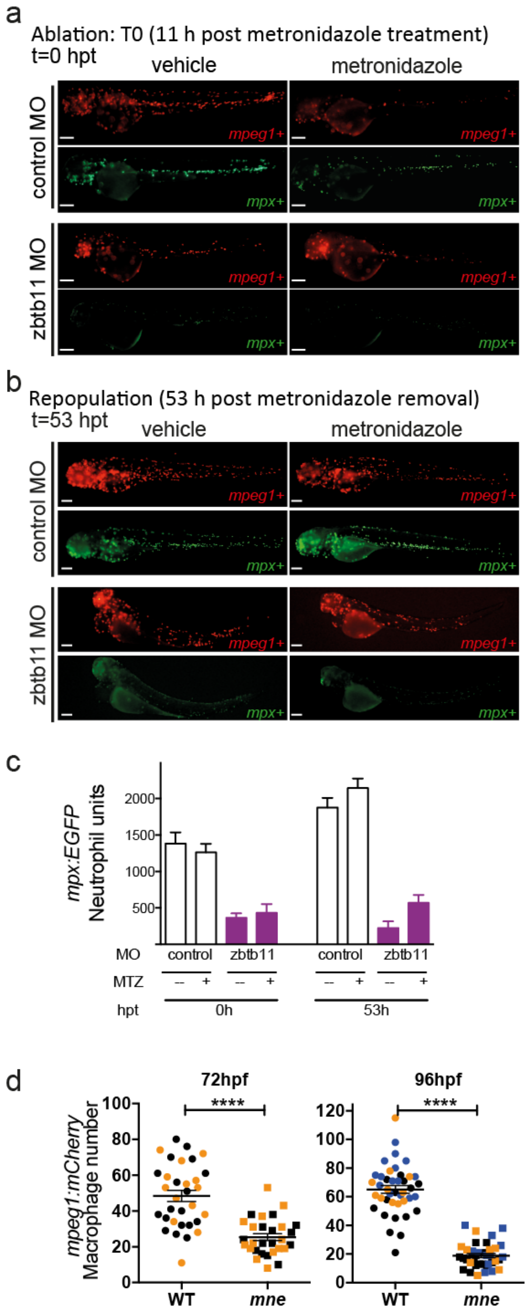

Supplementary Figure 4

Zbtb11 is dispensable for primitive macrophage development but is required for maintenance of macrophage populations in definitive haemopoiesis
(supports Fig. 3h)

a. Macrophages are ablated after 11 h treatment with metronidazole (t=0 hpt);
b. Macrophage repopulation occurs in both control and *zbtb11* morphants;
c. Neutrophils in the same animals and conditions as for **a** are unaffected by macrophage ablation (*n*=3 experiments; mean ±SEM);
d. Macrophages are depleted in *mne* at 72-96 hpf (*n*=3 experiments; each point represents an embryo; pooled data with embryos from individual experiments shown in different colours; 72 hpf, *n*=32,29; 96 hpf, *n*=41,38; mean ±SEM; Mann-Whitney test); panels **a, b** scale bar = 100 µm.

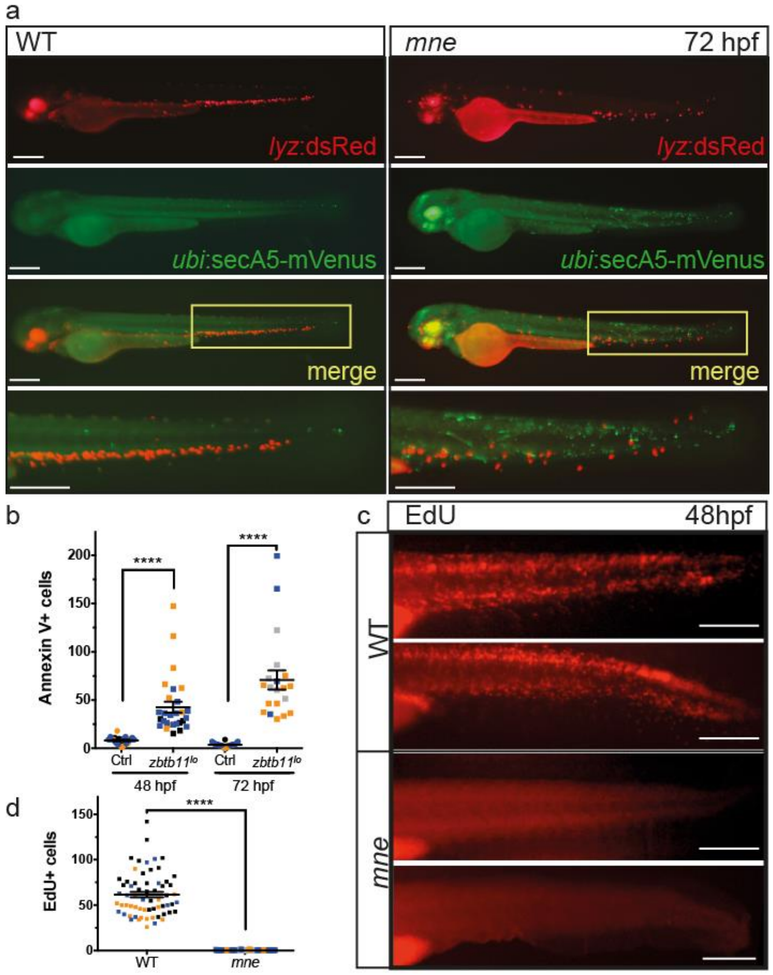

Supplementary Figure 5

Zbtb11 deficiency results in increased apoptosis and cell cycle arrest (supports Fig. 4)

- **a.** Overlay of *lyz*:dsRed and *secA5*:mVenus expression shows that apoptotic cells are not coincident with neutrophils in *mne*. Area in yellow box is expanded in lower panel;
- **b.** Quantification of apoptotic cells in zbtb11^{lo}. Pooled data with embryos from individual experiments shown in different colours (*mne* (*n*=1) or *zbtb11* morphants (*n*=2) and WT (*n*=3) experiments); from left to right: *n*=25,28,30,20; mean ±SEM; Mann-Whitney test);
- **c.** Representative images of EdU positive cells in the caudal haemopoietic tissue in WT and *mne*;
- **d.** Quantification of EdU positive cells in *mne* and WT; *n*=3 experiments, pooled data with embryos from individual experiments shown in different colours; WT, *n*=63; *mne*, *n*=73; mean ±SEM; Mann-Whitney test); panels **a, c** scale bar = 200 µm.

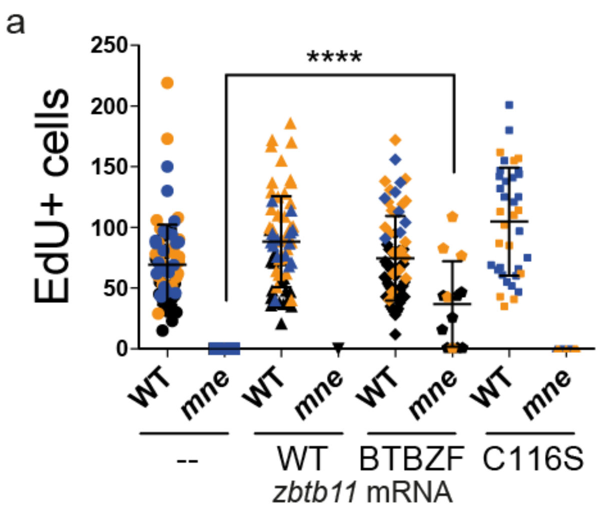

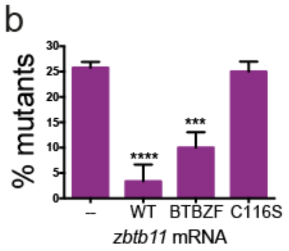

c

#	GT	EdU
3	*mne*	-
5	*mne*	+
4	WT	+
3	WT	+

Supplementary Figure 6.

N-terminally truncated Zbtb11 can rescue cell cycling in *mne*
(supports Fig. 5c)

- **a.** Enumeration of EdU positive cells in embryos injected with no (--), wild-type (WT), N-terminally truncated Zbtb11 (BTBZF) or C116S mutant Zbtb11 mRNA (C116S); *n*=3 experiments, pooled data with embryos from individual experiments in different colours; sample sizes are given for the total number of embryos per group and then for the number of embryos for each phenotype within the group. (--) *n*=116: WT=77, *mne*=39; WT Zbtb11 *n*=66: WT=65, *mne*=1; BTBZF *n*=83: WT=70, *mne*=13; C116S *n*=52: WT=37, *mne*=15; mean ±SEM; Mann-Whitney test; **** *p*=0.0001
- **b.** Percentage of mutants observed in clutches of injected embryos from a *mne* heterozygous in-cross (*n*=3 experiments; mean ±SEM; chi-squared analysis);
- **c.** Genotyping analysis of embryos overexpressing BTBZF mRNA. 10 embryos phenotyped as *mne* or partial *mne* and 5 embryos phenotyped as WT were randomly selected and all were molecularly genotyped. Correlation of genotyping with EdU staining identified 5/8 embryos genotyping as *mne* that showed EdU staining. Exact quantification of EdU staining is presented in **a.**

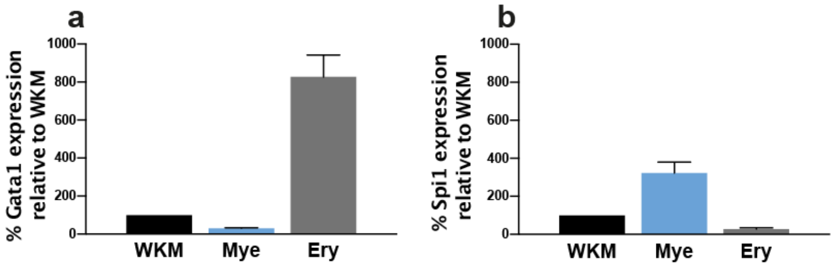

Supplementary Figure 7.

Confirmation of myeloid and erythroid population purity (supports Fig. 2e)

- **a.** RT-qPCR of Gata1 expression in FACS sorted adult zebrafish myeloid (Mye) and erythroid (Ery) cell populations compared to WKM (whole kidney marrow); *n*=1 in triplicate; mean ± SD;
- **b.** RT-qPCR of Spi1 (Pu.1) expression in FACS sorted adult zebrafish myeloid (Mye) and erythroid (Ery) cell populations compared to WKM (whole kidney marrow). *n*=1 in triplicate; mean ± SD.

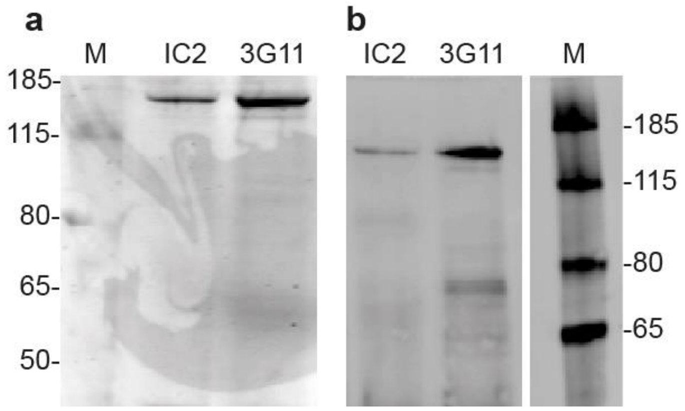

Supplementary Figure 8

Validation of IC2 anti-mouse ZBTB11 antibody
(supports Fig. 4f)

- **a.** Protein stain showing immunoprecipitation of mouse ZBTB11-GFP transfected into 293T cells by two anti-ZBTB11 rat monoclonal antibody clones, IC2 and 3G11. Immunoprecipitation was performed on 1 mg lysate with binding for 2 h in 4 μg/ml antibody followed by capture with Dynabeads (Thermo Fisher Scientific) at 4°C. Eluate was electrophoresed on a 4-12% denaturing polyacrylamide gel and stained with SimplyBlue SafeStain (Thermo Fisher Scientific). A single species is seen at ~140 kD;
- **b.** Immunoblot of immunoprecipitated ZBTB11-GFP with 0.5 μg/ml IC2 antibody, 2 h at RT, confirming identity of the protein bands. IC2 was used for ChIP assay as it appeared to have less background. M, protein ladder with molecular weight in kD as indicated.

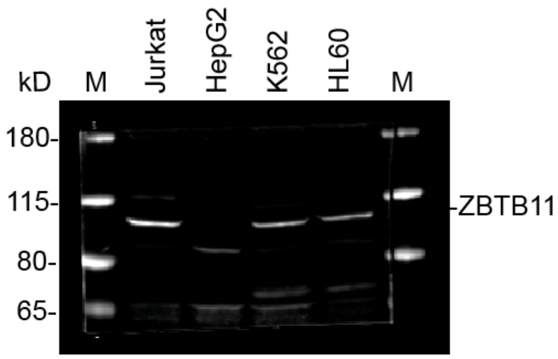

Supplementary Figure 9

Uncropped western blot
(supports Fig. 2f)

Immunoblot showing ZBTB11 is expressed in human myeloid and lymphoid cell lines and with lower expression in HepG2 hepatocytes (50 µg protein per lane); Membrane was cut to the size of the gel prior to protein transfer, and the uncropped image of the immunoblot is provided here. M, protein ladder with molecular weight in kD as indicated.

Supplementary Table 1 Primer Sequences

SSLP markers used for *mne zbtb11* positional cloning

SSLP	Primer F (5´-3´)	Primer R (5´-3´)
z22712	TCAAACGCATCAGACTGGTC	ACAGACGATCAGATCAGGGG
z9738	CAGATCCGGAGCTTCAACTC	CAGCCAGCAGTGTAAGCTGA
z20381	TGAAGAATGCACAAAGCCTG	TCCAACATCGACATACCAGC
z20932	TGTGGAAATGAGGAAAAGGC	CTGGTGTCAGACAGAGCCTG
z6767	AGGCCGGTAGCAATAATCAA	CCCTTCCGTATTGGGAATTC
z12094	ATCAGACCACAGACATGCCA	GCAATGACAGTTGGTTGACAA
z13538	CGTCCCGTCAAATATCATC	GTCTTTACATGGCTACATTGG
z25150	CCCCTTTGAGAAACAAGGGT	TGGGAAACACCCATACATTTT
z27076	CACTTGGCAGCGAACTATGA	GCTTGTAATGTTGCCCTCAAA
z55550	AGCGGGAAGGGAGATGTATT	GTTTGCACAGTGCACACAGT
z53452	ACAGAGCTGGGTGCATCTCT	TGGGTAGGCTAAATTCTCCG
z10183	CCGACATGGACAGACAATTG	TGTGTCCTGTTGGGGATACA
z5294	AGGGCGGCGAAATTGAAATT	AGCTCTAACCCCCTCCAAGCA
z7024	ATGGAGATGCTTCAACAGGG	CACACACATAAGGTGTTTGCA
z793	TGGTGAAATTTTGGACAGCA	CAAGGACCAAATGGAAGGAA
z22745	TGCTCAAACCAGCAACAGAC	CCCTCTGTTGACTAGTCCCC
z53172	GCTGTGGGGTGGTGAG	GGAGGCAGAGGTTGTGG
z8532	CGGCAGTTTATATGCAGCCT	CCTGAGACACAAGACAGCCA
z27232	GGCATATTCGTGTTTATCGTTG	TGTTGAGTTTTTCACATTTCAAATC
z21972	AGAGCTTTTAGCAGCCCCTC	CAGGGCTTCTTTGTTTCCTG
z13694	AGAGCTCAGAGGAACCCACA	CAGACGA TCCTTCA TCCTGG
z23904	GCTGCAA TCACCAGACTGAA	AACTGGCCTCTGTTACACGA
z20483	AACACAAAGTGCACACTACTGGA	CTTGCGGGTGCTATCTTTTC
z6495	ACCACCCACTTATCCAGCAA	TCAGTTGGCGTTTCTGTGTG
z25088	ATTTAGGTGTGTGCAGGGCA	AGAACTCACATCGTCAGGCC

RFLP and SSLP markers designed for *mne zbtb11* positional cloning

RFLP/SSLP	Primer F (5´-3´)	Primer R (5´-3´)
DC7	CTGACCCTCTTTGCTTCCAG	TTGCTGCAGAGATGGTTGTC
DC8	CCTGCTGGAGGAGTCGTAAG	GGCTGGACCAGTTGTCATTT
DC9	GGTAAAGTCCGTCCTCACCA	GGCCTCCAATCTCAATTCAA
DC10	ACCCACAAGCCACCACTAAG	TCAGGGCTGACTGAAGGTCT
DC11	CGGTGCATATGGACAGAGTG	ACATTGAGCGGAGAGGAAGA
DC12	CAGGCTCAATTCTCCTCTGG	ATGACAGCCAGCTTCTCCAT
DC13	GCCCATCAATCTTCCTCAAA	ACATTCGGTCCCAACAGAAG
DC18	ACCCAGCAATAGCGTCAGAT	GCCTGCATCTTATCCCAAAA
DC22	AGCTCGATTGGTGCAAAAGT	TTCTGCTGGCAATGAGTGAC
DC26	TGCTCGATCCATGTTGAACT	TGAAACCGTTTTGCTTTCCT
evi5 int 5	TCCTGACTTCAGGGTTTGTTG	TGTGAGGATTTTTACAAGATGAGG
rpl5b ex 3	ATTTGTGCACGTCTGCATTG	GCAGCATTGGCAAATATGGT
zbtb11 ex 2	TCTACTACTATCATAAGTACATGT TGC	CGCAAGTCTTCGTACATGAG
dpt exon 4	AACAACACACTGAGTCATTCG	GAAATGGCAATCAATGCAAG

Mutagenesis

H79A	GATCACGGAGGCGGCCATCACG TCCGGAG	CTCCGGACGTGATGGCCGCCTCC GTGATC
H86A	GTCCGGAGGGGAAGCCCTCAAC CAGCAG	CTGCTGGTTGAGGGCTTCCCCTCC GGAC
C119S	CAAGGAATGCATTCATAGCCAAA CCAAGCAGG	CCTGCTTGGTTTGGCTATGAATGC ATTCCTTG
N	GCAGTCCACTACAGTTGATTAAC ATGAACTGGTTTTTGTG	CACAAAAACCAGTTCATGTTAATCA ACTGTAGTGGACTGC
NBTB+	CTGAAGGAGCGGTGGAGTGAGA ACATGCGTGTAATG	CATTACACGCATGTTCTCACTCCAC CGCTCCTTCAG
NBTB (278)	GACCTTCAACTTTTGAGTGATGG	CAACCTCTGCCATCACGCAAAAG
S116C (to generate WT zf-zbtb11	GGACTGCATCAAGGAATGCATTC ATTGCCAAAC	GTTTGGCAATGAATGCATTCCTTGA TGCAGTCC
HBTBZF	GCCGAGAATTCAAAGGAACTGAT CACGGAGGCG	GCTCGACATTGAATTCGAATCGAT G
BTBZF	GAGGCGAATTCAGAGGAGCAGTC CACTACAG	GCTCGACATTGAATTCGAATCGAT G
BTB	CGAGGCGAATTCAGAGGAGCAGT CCACTACAG	GAGTTTCTAGATATGTTTCAGCAGC AGTGG
ZF	CATGCGAATTCAGAA GAGCAGAAGCTGATGG	GCTCGACATTGAATTCGAATCGAT G

Cloning

zbtb11 WISH probe	ATCGATGTCGAGCGAGGAGAGT	GTTTGGCAATGAATGCTTTCCTTGATGCAGTC
zebrafish *mne* zbtb11	GGATCGATGTTCCCCCTTGGAAAACATT	GGAGATCTGCAAACATTCACACATCCA
mouse Zbtb11	GTGCCGAATTCAATGTCAAGCGAGGAGAGCTACCGG	GACAGCTCTAGACACTCTGCCTCTGGCATATG
human ZBTB11	GGGAATTCATGTCAAGCGAGGAAAGCTACC	CCTCTAGACGTTACCAAACAGCCCAGA
zebrafish 2.3kb zbtb11 promoter	TTTCTCTATCGATAGGTACCCTACAATGGAGGGAGTGAA	CCGGAATGCCAAGCTTTAAATAACTCTCCTCGCTCG
human 2.9 kb ZBTB11 promoter	TTTCTCTATCGATAGGTACCGTGCATGTTACATTCAGACG	CCGGAATGCCAAGCTTTCGTTCGTCAGGTAACGC

TP53 qPCR ChIP

Primer set 1	AACCCCAATCCCATCAACCC	CAGGCGGATTACTTGCCCTT
Primer set 2	AGTAAGGGCAAGTAATCCGCC	TATCTACGGCACCAGGTCGG
Primer set 3	AGTCAGGATTCTCGCCGAC	TTTCCACCAATTCTGCCCTC
Primer set 4	TGTGAGGGCAGAATTGGTGG	AGCTGAGAGCAAACGCAAAAG

qPCR

zbtb11	AGAAAGGCTTGGATGAGTTC	TATTGTTTCTCCCCTTCTCC

Morpholino

zbtb11	AATAACTCTCCTCGCTCGACATTAC
tp53	GCGCCATTGCTTTGCAAGAATTG
standard control	CCTCTTACCTCAGTTACAATTTATA

sgRNA synthesis

zbtb11	GCACGCACGGCAACACCAAGAGG
